# Automatic detection of circulating tumor cells and cancer associated fibroblasts using deep learning

**DOI:** 10.1038/s41598-023-32955-0

**Published:** 2023-04-07

**Authors:** Cheng Shen, Siddarth Rawal, Rebecca Brown, Haowen Zhou, Ashutosh Agarwal, Mark A. Watson, Richard J. Cote, Changhuei Yang

**Affiliations:** 1 Department of Electrical Engineering, California Institute of Technology, Pasadena, CA 91125 USA; 2 Department of Pathology and Immunology, Washington University School of Medicine, St. Louis, MO 63110 USA; 3 Department of Biomedical Engineering, DJTMF Biomedical Nanotechnology Institute, University of Miami, Coral Gables, FL 33146 USA

**Keywords:** Cancer screening, Cellular imaging

## Abstract

Circulating tumor cells (CTCs) and cancer-associated fibroblasts (CAFs) from whole blood are emerging as important biomarkers that potentially aid in cancer diagnosis and prognosis. The microfilter technology provides an efficient capture platform for them but is confounded by two challenges. First, uneven microfilter surfaces makes it hard for commercial scanners to obtain images with all cells in-focus. Second, current analysis is labor-intensive with long turnaround time and user-to-user variability. Here we addressed the first challenge through developing a customized imaging system and data pre-processing algorithms. Utilizing cultured cancer and CAF cells captured by microfilters, we showed that images from our custom system are 99.3% in-focus compared to 89.9% from a top-of-the-line commercial scanner. Then we developed a deep-learning-based method to automatically identify tumor cells serving to mimic CTC (mCTC) and CAFs. Our deep learning method achieved precision and recall of 94% (± 0.2%) and 96% (± 0.2%) for mCTC detection, and 93% (± 1.7%) and 84% (± 3.1%) for CAF detection, significantly better than a conventional computer vision method, whose numbers are 92% (± 0.2%) and 78% (± 0.3%) for mCTC and 58% (± 3.9%) and 56% (± 3.5%) for CAF. Our custom imaging system combined with deep learning cell identification method represents an important advance on CTC and CAF analysis.

## Introduction

Approximately 90% of all cancer-related deaths are due to the process of metastatic spread^1,2^. During metastasis, tumor cells detach from the primary tumor, migrate into blood vessels and these circulating tumor cells (CTCs) travel to distal organs through the circulatory system. CTCs tend to survive even in the harsh circulatory environment and retain the ability to proliferate and create secondary tumor sites in distant organs^3,4^. Over the past decades, there has been increasing interest in understanding the metastatic cascade with respect to how CTCs gain access to the circulatory system, survive in circulation, and thereafter create secondary metastatic sites, as well the biology behind tropism towards distant organ sites for different tumor types^5–9^. This has brought CTCs to the spotlight in a rapidly expanding field of liquid biopsy and cancer diagnostics.

CTCs can be found as either single cells or clusters, with the latter being formed by tumor cells alone or more significantly, as clusters of multiple cell types such as peripheral blood mononuclear cells (PBMCs) and circulating cancer associated fibroblasts^10–12^. It has become increasingly clear that in addition to CTCs, the circulating cells in the tumor microenvironment (TME) also play an important role in metastasis. Cancer-associated fibroblasts (CAFs) are a group of activated fibroblasts with significant heterogeneity and plasticity in the TME^13–16^. It has been shown that CAFs also gain access to the circulatory system and play a significant role in the process of metastasis. They modulate cancer metastasis through synthesis and remodeling of the extracellular matrix (ECM) and production of growth factors. Thus, targeting CAFs has gained considerable interest and is being explored as an avenue for novel cancer therapies.

In general, CTCs are extremely rare in blood samples and range from one to a hundred cells in a 7.5 mL tube of human blood, depending on the stage of the disease^9^. CTC clusters are even rarer and constitute only 2–5% of all CTCs^17^. However, CTC clusters are disproportionately efficient at seeding metastases. Therefore, CTCs and CTC clusters are valuable biomarkers to determine prognosis, monitor therapy, assess risk of recurrence, and possibly in the future to aid in early cancer detection and screening^3–12^. Over the past decade, numerous CTC capturing techniques have been developed^17–20^. The different enrichment and isolation processes either leverage the morphological characteristics of CTCs which include size/deformability-based separation^21–23^ and density-gradient centrifugation^24,25^, or rely on immunoaffinity-based separation through targeting specific cell surface epitope expression^26,27^. The identification and characterization of CTCs can be performed in several ways: immunofluorescence^28^, fluorescence in situ hybridization (FISH)^29^, real-time reverse transcription polymerase chain reaction (qRT-PCR)^30^, genomic analysis^22^, and RNA sequencing^31^. Capturing of CAFs can also be achieved through similar methods used for CTC^14,32,33^. Our team has developed a size-based enrichment technology using a membrane microfilter device with defined pore size to isolate CTCs and CAFs from whole blood as well as other bio-fluids^34^. Exploiting the size characteristics of CTCs and CAFs (being larger than the vast majority of normal blood cells), the pores allow the smaller blood cells to be filtered through while capturing the larger CTCs and CAFs along with some extraneous, larger sized mononuclear cell lineages. Among the methods discussed above, size-based isolation techniques combined with immunofluorescence labelling have gained significant traction due to the simplicity of this technique, its unmatched capture and enrichment efficiency, and its ability to capture and enrich CTC of different embryologic origin (e.g., epithelial vs. mesenchymal), because size-based filtration of CTC does not rely on the immunoaffinity methods.

This approach has two technical challenges that are the focus of our present study. The first challenge is that while the microfilter membranes (approximately 15 microns thick) appear to be flat and uniform, they are flexible and have slightly wavy surfaces, thus presenting widely varying focal planes. The small height variations of these surfaces will be greatly magnified under the high powers of microscopy needed to identify and characterize CTC and other captured cells. Since the captured cells are randomly distributed across the wavy microfilter surface, they are, in turn, distributed across different focal planes during microscopic imaging. This makes it difficult to utilize commercially available slide scanners to collect whole filter scans with all cells in-focus and requires robust auto-focusing ability of the imaging system across not only the different fields-of-view (FOVs) but also even within the same FOV. Commercial scanners are ill-suited to handle this degree of focal plane heterogeneity^35^, especially within the same FOV. Currently, the only way to address this is to acquire multi-focal-plane data (‘*Z*-stacking’) for each FOV so that all cells within each FOV can be digitally focused post-imaging.

Second, detection of CTCs and CAFs on the microfilter is currently a manual process. The user needs to evaluate a large number of captured cells to identify the very few that are of interest (CTCs, CAFs), and these cells are often morphologically similar to other captured cells, making the analysis of these filters laborious, inefficient, and subjective. Automating the cell localization and classification process has been attempted by employing image processing and machine learning (ML) algorithms on fluorescent microscope images of CTCs^36–39^. The general workflow for such a process requires several steps. Initially, a region of interest (ROI) identification algorithm is designed to segment all possible CTC or CAF events out of the whole slide images^39^. Then the pre-screened image patches are normalized and transformed to a feature space by human engineering. For example, the features could be pixel gray value histograms in each red–green–blue (RGB) channel^36^ or vector arrays in hue-saturation-value (HSV) color space^37^ or morphological characteristics including area, perimeter, and eccentricity^38^. Finally, these feature representations of events are classified by conventional machine learning models, such as naive Bayesian classifier^36,38^, support vector machine^36–38^ or random forest^37,38^. This type of cell detection workflow is not holistic, and the accuracy of upstream steps will inevitably affect the performance of downstream ones as detection errors accumulate. In addition, these methods are, at best, semi-automated, as they heavily rely on manual input, such as the parameter tuning for segmentation process and the manual decision on feature engineering. We believe that deep learning (DL) is a better method, as it has the advantages of enabling self-learned feature engineering and end-to-end training. To date, only a few studies^40,41^ have explored applying DL for CTC analysis, and there are none so far on the use of DL for CAF analysis.

In this study, we have addressed both challenges and presented a comprehensive scheme to automatically detect and analyze tumor cells and CAFs that are captured by our microfilter device. We utilized a model system of cultured cancer cells to mimic the processing of a clinical sample but in a controlled fashion. We refer to these cultured tumor cells as mimicked CTCs (mCTCs). First, we have developed a hardware imaging solution that can collect high quality, uniformly in-focus images. Second, we have used these high-quality in-focus images for deep learning model training and subsequent analysis of new samples using DL.

## Results

**Figure 1:**
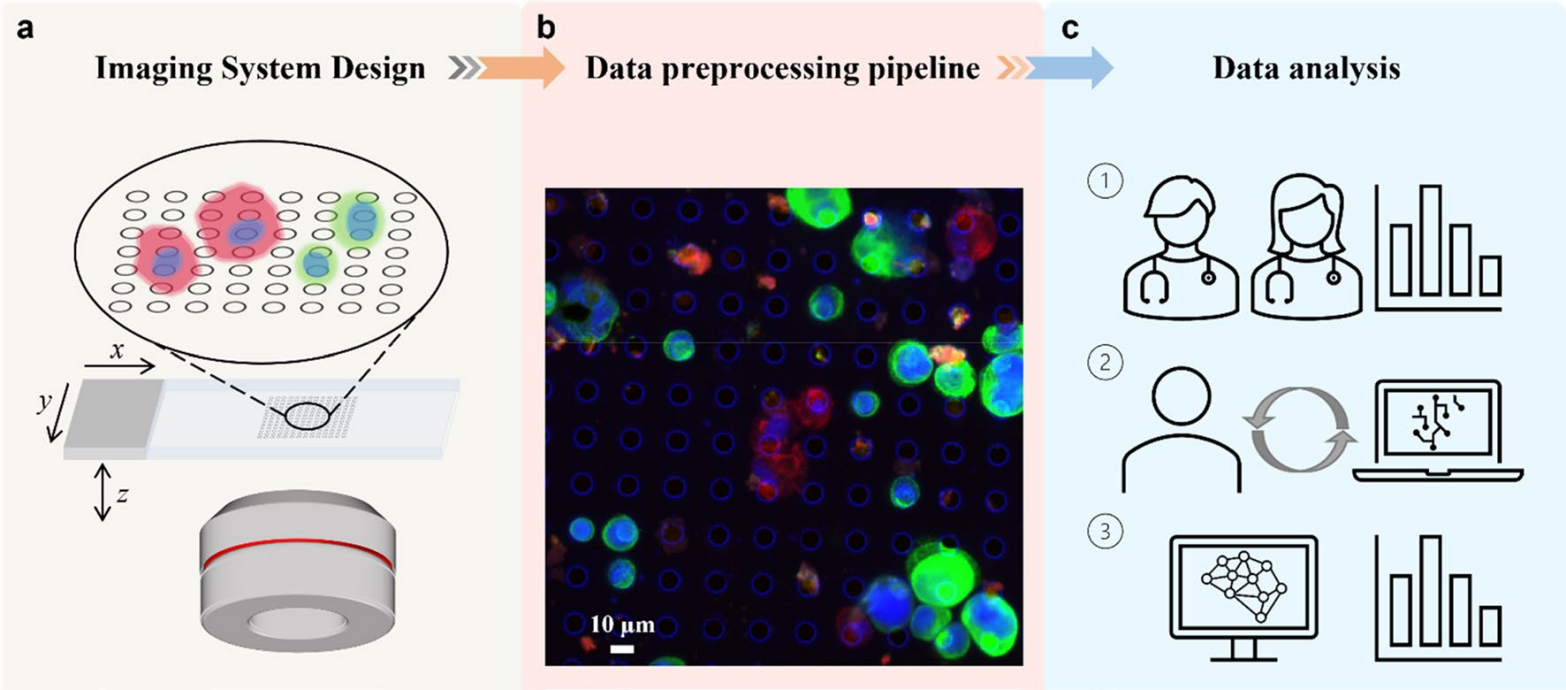
Schematic of overall design. (**a**) Multi-channel epifluorescence microscope imaging system. Since our target cells are distributed on the micro-filter at varied heights, the sample is three-dimensional in nature. They are scanned axially under four channels to fully capture the cell-specific biomarker expression. (**b**) Data preprocessing pipeline. The raw image data are synthesized into a single multi-color all-in-focus whole slide image for further analysis. (**c**) Data analysis. The classical way to detect CTCs and CAFs relies on human experts. ① First, the experienced pathologists review the whole slide, annotate cells of interest, and count their number. ② Then this annotation paired with fluorescence images is used to train a deep learning model. Because of inherent human observer bias in calling or ignoring positive cells, the prediction from the pre-trained deep learning model is used to cross-validate human expert annotation. ③ Finally, the well-trained deep learning model can independently conduct the cell detection and analysis task.

The overall approach of this work consists of three stages depicted in Fig. 1: (a) Autofocusing and axial scanning design of our customized microscope system to ensure that information of all cells located at different focal planes can be acquired. (b) An optimized data preprocessing pipeline to synthesize raw multi-channel multi-slice images into a single all-in-focus (AIF) RGB whole slide image for each sample. (c) A collection of these AIF slide images that are annotated by human experts and subsequently used to train deep-learning-based mCTC and CAF detection systems. The trained systems thereafter can be used to automate the analysis of new samples.

### Slide scanner with better auto-focusing ability

**Figure 2:**
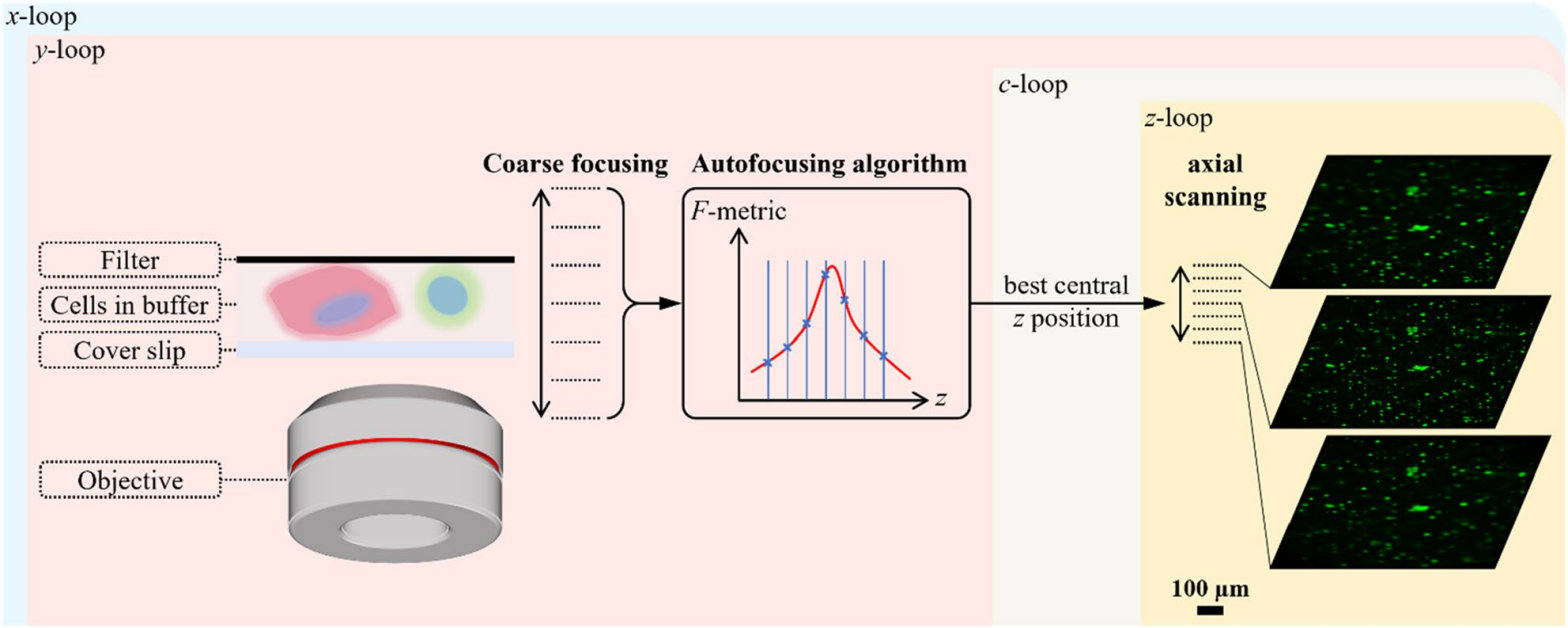
Auto-focusing principle during scanning. First, a coarse scanning with large step size over a wide *z* range is performed. Then, the image at each *z* position is used to calculate the focus measure (*F*-metric). The best focus *z* position is then estimated as the peak location by fitting a Gaussian function to discrete *F*-metrics. Centered on this estimated best focus *z* position, a fine axial scanning with small step size is performed to capture the whole 3D information. Autofocusing is repeated for every lateral *x*–*y* scanning position and executed only in DAPI channel. The estimated best focus *z* position will be used across all channels. Chromatic aberration can be compensated by axial scanning.

Commercially available slide scanners that host autofocusing functions have been unable to provide a uniform in-focus whole scan of our microfilter. Without the ability to acquire AIF whole scans, it is difficult to analyze clinical samples with high accuracy and confidence and at times unable to analyze them at all. Therefore, we set out to emulate the manual focusing process with a customized scanning strategy for an epifluorescence microscope. As shown in Fig. 2, we started this process by performing coarse focusing with a large *z* step size over a large range. Next, we calculated the focus measure metric (*F*-metric) based on this *z* stack. There have been extensive studies on different types of autofocus metric functions for fluorescence microscopes^42^. For our purpose, we used the Tenegrad function—a robust and simple *F*-metric (see Supplementary Fig. S1 for more details on this selection choice). To achieve an accurate estimate of the best focus position, we fitted a Gaussian function to the discrete *F*-metric sequence from *z*-stack and took its peak as the final best focus position, with which we further conducted the fine axial scanning with small *z* step size. Through fine scanning, cells located at different focus planes can be digitally revisited afterwards or synthesized into a single 2D AIF image.

For our system, an Olympus achromatic objective lens (PLN 20X) with a numerical aperture (NA) of 0.4 was used and its depth of field was 1.72 μm. The coarse *z*-axis focusing step size was 20 μm with a scan range of 200 μm and the fine scanning *z* step size was 5 μm with a scan range of 50 μm.

We can apply this strategy to image a large sample by repeating the process for overlapping FOVs across the entire sample by lateral scanning. The best *z*-focus estimation only needs to be run once for each lateral position and thereafter is applied across all the fluorescence channels. The number of fluorescence channels utilized is dependent on the immunofluorescence staining protocol for the biomarkers used for mCTC and CAF identification (see "Methods"). Residual axial chromatic aberration from the objective can be addressed when we process the fine-step *z*-stack data collected in the second step.

### Data preprocessing pipeline to generate all-in-focus whole slide image

**Figure 3:**
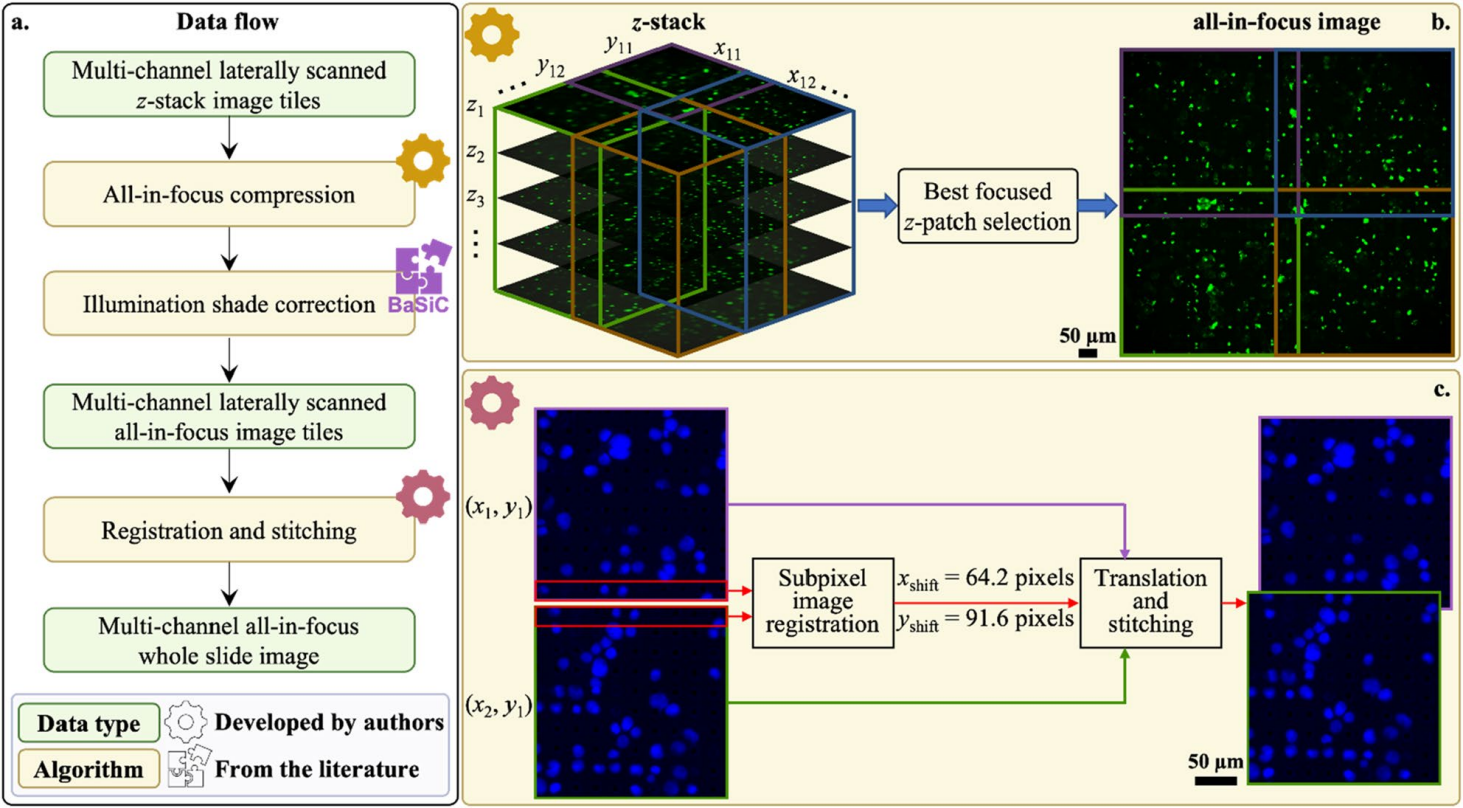
Data preprocessing pipeline. (**a**) Data flow starting from raw measurement and ending with a multi-channel all-in-focus whole slide image. Preprocessing consists of three algorithms, among which two are developed by authors and the other one is adapted from an existing work. (**b**) Principle of all-in-focus compression. *Z*-stack at each *x*–*y* location is split into smaller patches and the best focused *z*-patch is selected with focus measure. Finally, *z*-patches are fused into an all-in-focus *x*–*y* tile. **c** Principle of registration and stitching. There is overlap between adjacent *x*–*y* tiles due to the tilt between scanner lateral movement coordinates and camera frame coordinates. Subpixel image registration algorithm relies on the overlapping region to find the subpixel shift between two adjacent *x*–*y* tiles. Taking the upper left corner tile (*x*_1_, *y*_1_) as the anchor for the final mosaic, all other *x*–*y* tiles are translated and stitched to it by blending based on distance transform.

For each microfilter slide, we acquired multi-channel laterally scanned *z*-stack image data by the above process. Our end goal was to render a single AIF RGB whole slide image so that it can be effectively processed by the subsequent DL model without defocus-associated inaccuracies. As a sidenote, this single AIF whole slide image is also a good fit for pathologist’s workflow as it eliminates the need to manually retune the image focus during analysis. In our experiment, this rendering process consisted of three steps (see Fig. 3).

First, we compressed the images’ *z*-dimension by cropping the image tile for a single FOV into smaller patches with the size of 600 × 600 pixels and selecting the *z*-slice with highest focus quality for each patch. (The patches were partitioned in such a way that there is some overlap with neighboring patches.) This allowed us to flexibly put cells in the single FOV, which may be located at different *z*-planes, into focus. We again adopted the Tenegrad function in selecting the best focus quality. As the collected fluorescence image can be affected by non-uniform excitation light profile due to vignetting effect and temporal background variation, we next normalized the image brightness spatially. To accomplish this, we used an established shade correction method, BaSiC^43^. Finally, we used a customized registration algorithm and leveraged the overlap between neighboring patches to stitch these patches together into the entire whole slide FOV. This customized stitching algorithm was necessary to accommodate for tilts between the lateral movement coordinates of the motorized scanner and the camera frame, which prevented a simple direct stitching of the patches. For our experiment, we typically acquired a whole slide FOV of size 6.9 mm × 6.9 mm. Our objective allowed us to acquire single image tile FOVs that were 0.92 mm × 0.85 mm, corresponding to 4000 × 3700 pixels. Between two neighboring patches, the overlap was 310 pixels.

**Figure 4:**
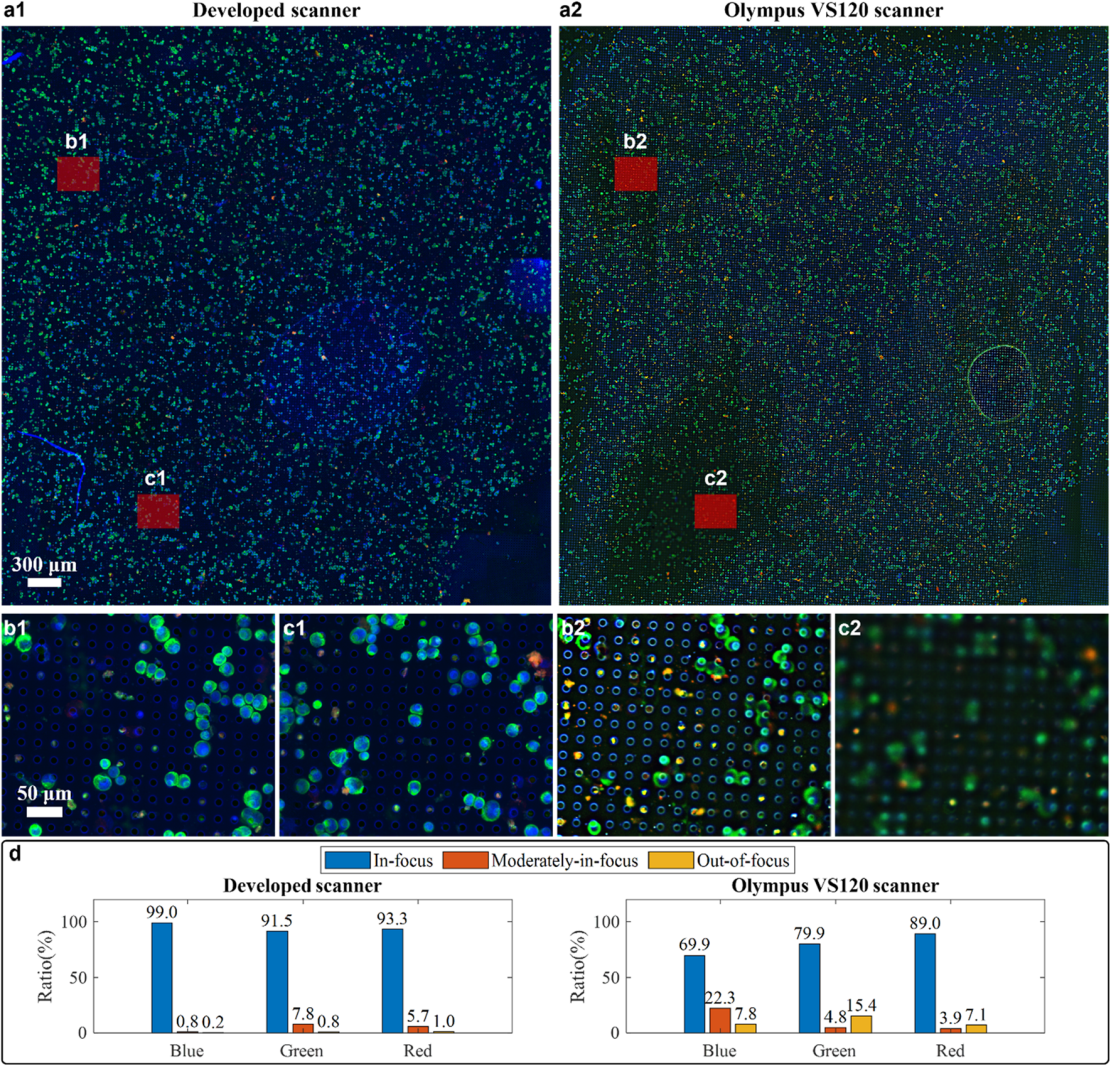
Comparison of the whole slide image focus quality by our developed scanner and Olympus VS120 scanner. (**a1**) Whole slide image (WSI) of a model sample under 20X objective from our developed scanner. (**a2**) WSI of the same model sample under 20X objective from Olympus VS120 scanner. (**b1**,**b2**) and (**c1**,**c2**) are the zoom-in on the same regions from two WSIs. Their area size is the same as the image tile from VS120 scanner, 366 μm × 287 μm. (**d**) Quantitative analysis of focus quality of WSI from both scanners in blue, green and red channel.

This combination of optimized auto-focusing and image processing algorithm created a whole slide image of the microfilter with high focus quality. To verify its performance, we compared our system with a commercial slide scanner, Olympus VS120, which we have used in the past to perform whole microfilter scans (Fig. 4). The same sample was scanned using both our custom developed scanner and the Olympus VS120 scanner. Two whole slide images were registered, and the maximum overlapping region was displayed. From Fig. 4a, we can clearly see that our developed system was able to achieve a more uniform focus quality than the Olympus scanner. The commercial scanner only performs focus selection at the image tile level (nominally at an area size of 366 μm × 287 μm) while our system performs focus selection at a smaller patch level (nominally at an area size of 138 μm × 138 μm). Moreover, the commercial scanner was unable to correctly focus over a significant fraction of the sample, as can be seen in areas noted in Fig. 4b,c.

To quantify the AIF performance difference in this characterization experiment, we utilized a deep neural network model that predicts an absolute measure of focus quality at a patch level for fluorescence images^44^. The model was originally trained on wide field fluorescence images of U2OS cells with Hoechst-stained nuclei. It has demonstrated robust generalization capabilities to other stains, such as Tubulin and Phalloidin, as well as the MCF-7 cancer cell type, despite not being explicitly trained on them. Given that our images also involve wide field fluorescence imaging of cancer cells, we anticipate that this method should generalize well to our dataset as well. This model outputs a probability distribution over 11 discrete focus levels for an 84 × 84 pixel patch; the focus level with highest probability is taken as the final prediction. We ran this model, patch by patch, over each channel of two whole slide images and generated a focus quality map with the granularity of 84 × 84 pixels. For better visualization, a bar chart to summarize the portion of patches with different focus quality for two WSIs is shown in Fig. 4d. Here, the first three focus levels are binned into ‘In-focus’ class, the middle four focus levels are binned into ‘Moderately-in-focus’ class and the last four focus levels are taken as ‘Out-of-focus’ class. In order to ensure that the predicted focus quality measures align with human vision evaluation, we conducted a manual verification. Our visual evaluation indicated good agreement between the predicted focus quality measures and the actual images. Specifically, the patch images classified as 'In-focus' showed clear cell cytokeratin details, while the 'Moderately-in-focus' images still display recognizable cell cytokeratin features. In contrast, the 'Out-of-focus' images barely resembled cells and often resembled precipitates. We have provided several examples of each class in Supplementary Fig. S2. Our custom scanner performed with an average 99.3% of regions across different channels in the WSI either in-focus or moderately in-focus. In contrast, the in-focus or moderately-in-focus portion only occupies an average of 89.9% area of WSI from the Olympus scanner. This is especially problematic for our application because almost 10% of the Olympus scanner image was out-of-focus—a data loss that is undesirable for our purpose.

As a sidenote, the defocus output from the model can be associated with the physical defocus of our system. In^44^, it was concluded that the trained model can generalize on previously unseen fluorescence images, identifying the absolute image focus to within one defocus level (approximately 3-pixel blur diameter difference) with 95% accuracy. According to our system parameters and considering the size of mCTCs is larger than 8 μm, we established that our ‘in-focus’ class corresponds to target objects within ± 2.5 μm of the focus, the ‘moderately-in-focus’ class corresponds to target objects between ± 2.5 μm and ± 7 μm of the focus, and the ‘out-of-focus’ class corresponds to all distances beyond.

### An ensemble deep learning approach for detecting mCTCs and CAFs

We next implemented an automated cell detection and identification system by training a deep learning network on the data collected with our imaging system. To obtain a large number of training instances, we prepared model sample slides using specific cell lines (see Model system slide preparation in Methods). Then, the WSIs from these model system slides were split into training and testing datasets at the level of individual slides. Additional information on the data description and splitting scheme is presented in Supplementary Fig. S3.

**Figure 5:**
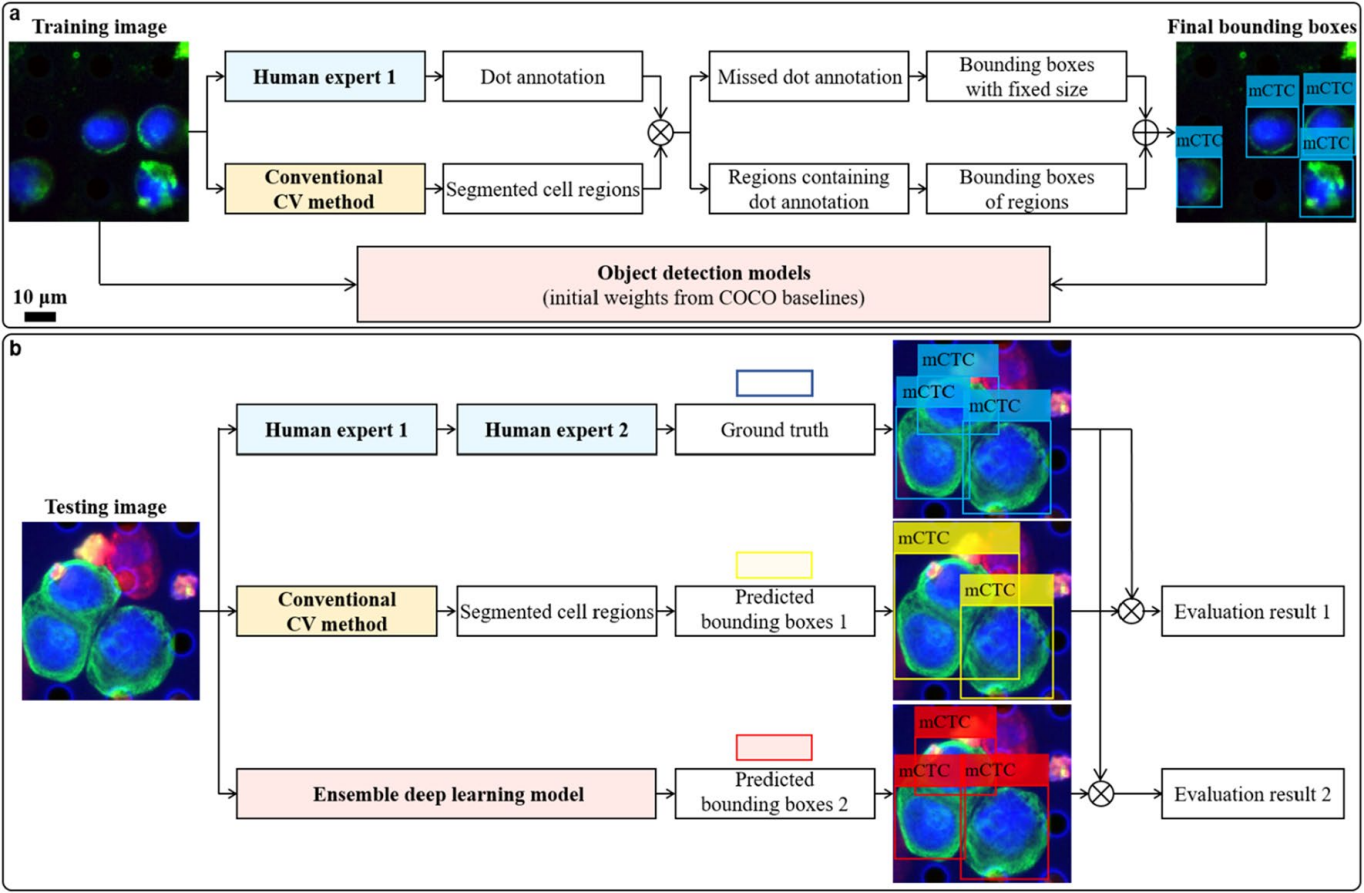
Cell detection via deep learning. (**a**) Training pipeline. An experienced pathologist annotates the cells of interest in training images with dots and simultaneously these images are processed by a conventional computer vision (CV) method to segment cell regions. Results from both methods are cross validated by matching annotation dots and segmentation regions. Any region containing annotation dots is used to generate a bounding box and paired with the annotation label. For dots which do not lie in any region, a bounding box centered at each of them is generated with the size of empirical cell diameter. Then, training images and their corresponding bounding boxes with class labels are used to train a generic object detection deep learning model. Here, transfer learning is adopted by using weights pretrained on the COCO benchmark dataset. (**b**) Testing pipeline. The unseen testing images are analyzed in three ways. First, the same experienced pathologist screens testing images by annotating the cells of interest with bounding boxes, which are sequentially double checked by another computational pathology researcher to make sure there is no oversight or mislabeling. This result is taken as ground truth. In parallel, testing images are segmented by the conventional CV method and then the prediction boxes with labels are generated from segmented regions. Finally, they are sent to our well-trained cell detection model and the predicted bounding boxes can be directly generated. Comparing results from the latter two methods with the ground truth, we find our trained deep learning model outperforms the conventional CV method.

The scheme for cell detection via deep learning is summarized in Fig. 5. For the training image set, the cells of interest were manually annotated in QuPath^45^ using the dot annotation function for fast screening. However, the dot format was insufficient to generate the bounding box for each cell. Therefore, a conventional computer vision (CV) method based on pathologist screening protocol and image processing algorithms was also developed to coarsely detect cells of interest (see details in Supplementary Figs. S4 and S5). Results from both methods were cross validated by matching annotation dots and segmentation regions. Any region containing annotation dots was used to generate a bounding box and paired with the annotation label. For dots outside any region, bounding boxes that centered them were generated with the side length matching the empirical cell diameters reported in previous studies. Then, training images and their corresponding bounding boxes with class labels were used to train a generic object detection DL model. To reduce the training time and simplify hyper-parameter tuning process, transfer learning was adopted by using weights pretrained on the COCO benchmark dataset^46^. Furthermore, to decrease model performance variation, we ensembled the predictions from five models with the same architecture that had been separately trained on different image batches, as shown in Supplementary Fig. S6. This process was executed for mCTCs and CAFs separately.

Our choice of DL model was informed by the following process. The varieties of neural network architectures for object detection can be categorized into two groups, one-stage detectors and two-stage detectors^47,48^. Their respective representatives are RetinaNet^49^ and Faster R-CNN^50^. We paired them with different backbones, ResNet-50^51^, ResNet-101^51^ and ResNeXt-101^52^, as well as pre-trained weights on different benchmark datasets, including COCO^46^ and Pascal VOC^53^. The precision-recall curves of different models for mCTC detection are summarized in Supplementary Fig. S7a using fivefold cross validation. From that analysis, we saw that RetinaNet and Faster R-CNN with ResNet-101 backbone pretrained on COCO dataset achieved the best area under curve (AUC) in one-stage and two-stage detectors, respectively. In general, RetinaNet models worked better than Faster R-CNN models. Therefore, we finally chose the RetinaNet model for the mCTC detection task. Based on this result, we only tested the RetinaNet and Faster R-CNN with ResNet-101 backbone pretrained on COCO dataset on the CAF detection task. We experimentally found that Faster R-CNN has better localization and recognition accuracy, as indicated by the higher AUC in Supplementary Fig. S7b. As such, we chose Faster R-CNN for the CAF detection task.

The conventional CV method to detect mCTCs and CAFs from RGB fluorescence images was designed based on the pathology screening protocol shown in Supplementary Fig. S4. Its pipeline is summarized in Supplementary Fig. S5. This method does not rely on any conventional machine learning techniques such as support vector machine. First, three channels were separately binarized to identify numerous positive events, DAPI positive (blue channel), cytokeratin (CK) positive (green channel) and fibroblast activation protein (FAP) positive (red channel). Then, CK and FAP positive events were segmented using watershed transform and cleaned by removing event regions with size below or beyond expectations. Finally, binary images from each of the three channels were cross-checked. The event regions which were DAPI and CK positive but FAP negative were accepted as CTCs and those which were DAPI and FAP positive but CK negative were identified as CAFs. Here, DAPI positive events got confirmed by calculating mean intensity value within the area of CK/FAP positive events. This approach helped reject most of the microfilter holes due to their hollow structure. During this image processing pipeline, the binarization threshold, segmentation parameters and size threshold were all chosen and optimized by trained observer guidance.

To evaluate the cell detection performance of our system on unseen test slide samples, we designed the testing phase of the experiment as follows. First, the same experienced pathologist screened testing images by annotating the cells of interest with tight bounding boxes, which were consecutively double checked by a second reviewer to make sure there was no oversight or mislabeling. This result was taken as the ground truth. Then, testing images were segmented by the conventional CV method in the same way as training images. On a parallel track, we also used our trained ensemble DL model to directly predict bounding boxes on the testing images. The ensemble strategy is illustrated in Supplementary Fig. S6. Comparing the results from the two computational methods with the ground truth, we found our final ensemble DL model significantly outperformed the conventional CV method.

### Ensemble deep learning model outperforms conventional computer vision method

**Figure 6:**
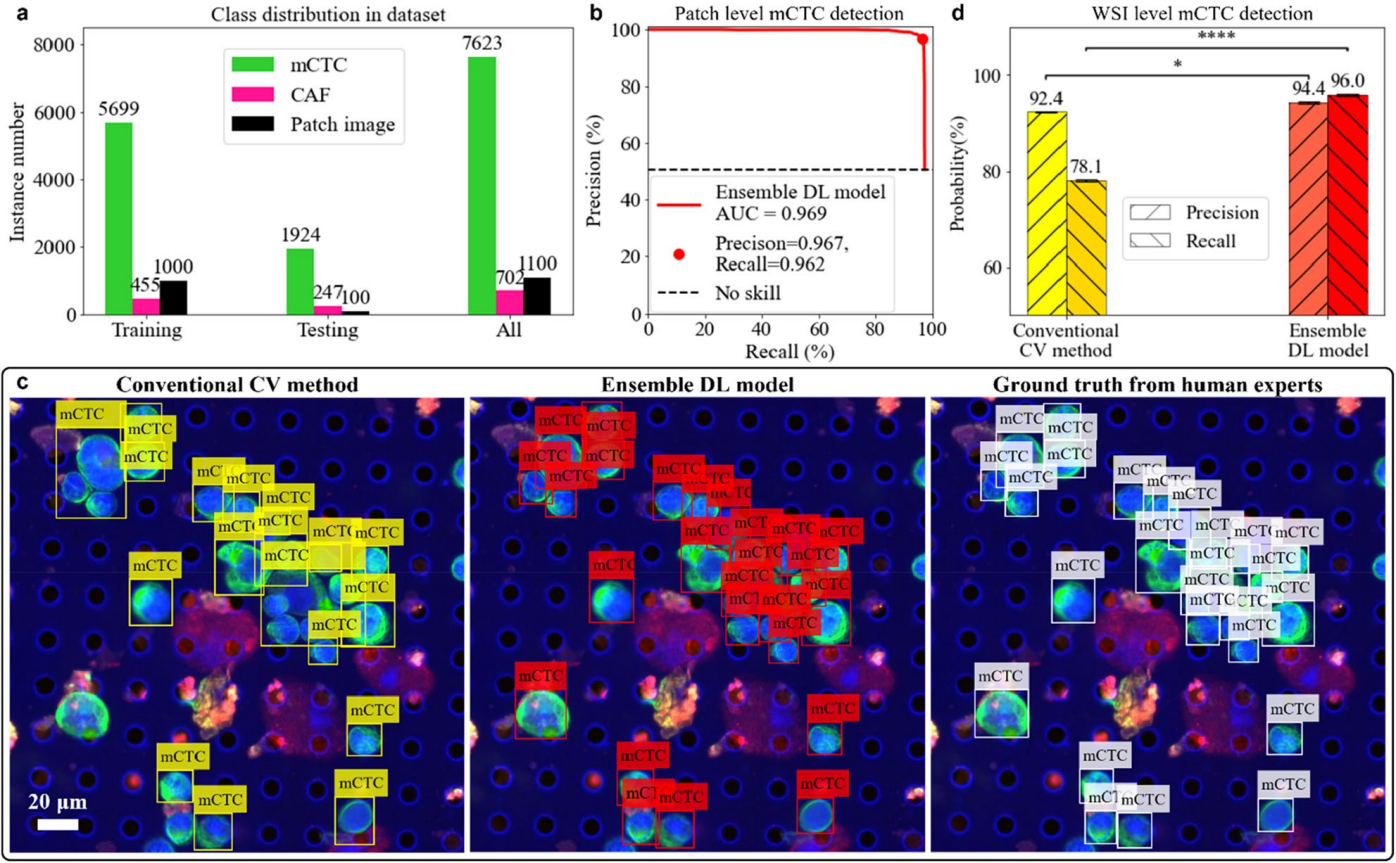
Evaluation of mCTC detection. (**a**) Class distribution and the number of patches images in the training/testing/whole dataset. (**b**) Precision-recall curve of the ensemble deep learning (DL) model to detect mCTCs in testing patch images. The red dot represents the final chosen operating point. (**c**) Example of mCTC detection by conventional computer vision (CV) method and ensemble DL model shown horizontally with ground truth from human annotation. (**d**) Performance comparison between conventional CV method and ensemble DL model to detect mCTCs on the whole slide image level. Both precision and recall of ensemble DL model are significantly higher than the ones of conventional CV method. Statistical analysis uses the ensemble DL model result as the reference to test their difference significance, error bars show standard deviation of precisions and recalls by randomly sampling testing dataset 1000 times and the *p*-values are specified in the figure for *p < 0.05, **p < 0.01, ***p < 0.001, ****p < 0.0001, NS, not significant, two-sided *z*-test.

The output of the DL detection model for a testing image patch was a set of bounding boxes with confidence scores. As shown in Supplementary Fig. S6, the bounding boxes with scores below the threshold were discarded. By adjusting the threshold to this score, we altered the precision and recall of our ensemble model, shown as the precision-recall curve in Fig. 6b. The AUC was 0.97 for mCTC detection—a good indication that our ensemble model performed well. To balance type I and type II errors, the final operating point was chosen to give precision of 97% and recall of 96%. It is worth noting that the input image size used for both training and testing in our study was 1000 × 1000 pixels, which was cropped from much larger whole slide images. In cases where mCTCs were located on the boundary of the FOV, only a portion of the cell would appear within the DL model's FOV at any given time. For instance, in some cases, only part of the CK signals would be visible without any DAPI signal. To avoid any ambiguity in our testing and ensure accurate cell counting, we only annotated and tested cells that were completely visible within the FOV of patches and excluded those that were partially visible. When the ensemble model was directly applied to the WSI, it tended to miss the cells lying on the patch image boundary. If we were to naively apply our trained model, the precision and recall for detecting mCTCs at the WSI level would drop to 93% and 94%, respectively. To solve this problem, we proposed a whole slide image detection scheme described in Supplementary Fig. S8. The patch coverage was shifted horizontally and vertically by the half size of DL model input FOV. Then repeated detections were removed by calculating the overlapping ratio between each two of the predicted bounding boxes. After accounting for this, we obtained a final precision and recall at the WSI level of 94% (± 0.2%) and 96% (± 0.2%), respectively. In contrast, when we directly applied conventional CV method on the WSI, we obtained a precision of 92% (± 0.2%) and a recall of 78% (± 0.3%). In general, the conventional CV method can be seen as a conservative detector with precision close to our ensemble DL model but is more likely to miss the mCTCs.

Figure 6 shows an example of mCTC detection by an experienced pathologist, by the ensemble DL model, and by the conventional CV method. We can see that conventional CV method missed a significant number of mCTCs for two major reasons. First, the conventional image segmentation method appeared to have difficulties segmenting clumps of cells. It mistakenly predicted several mCTCs as one. Second, mCTCs that were surrounded by precipitates with other stains appeared to be more likely to be eliminated after thresholding in other channels. Our ensemble DL model neither down-segmented the mCTCs nor was it strongly affected by precipitates attached to the mCTCs.

**Figure 7:**
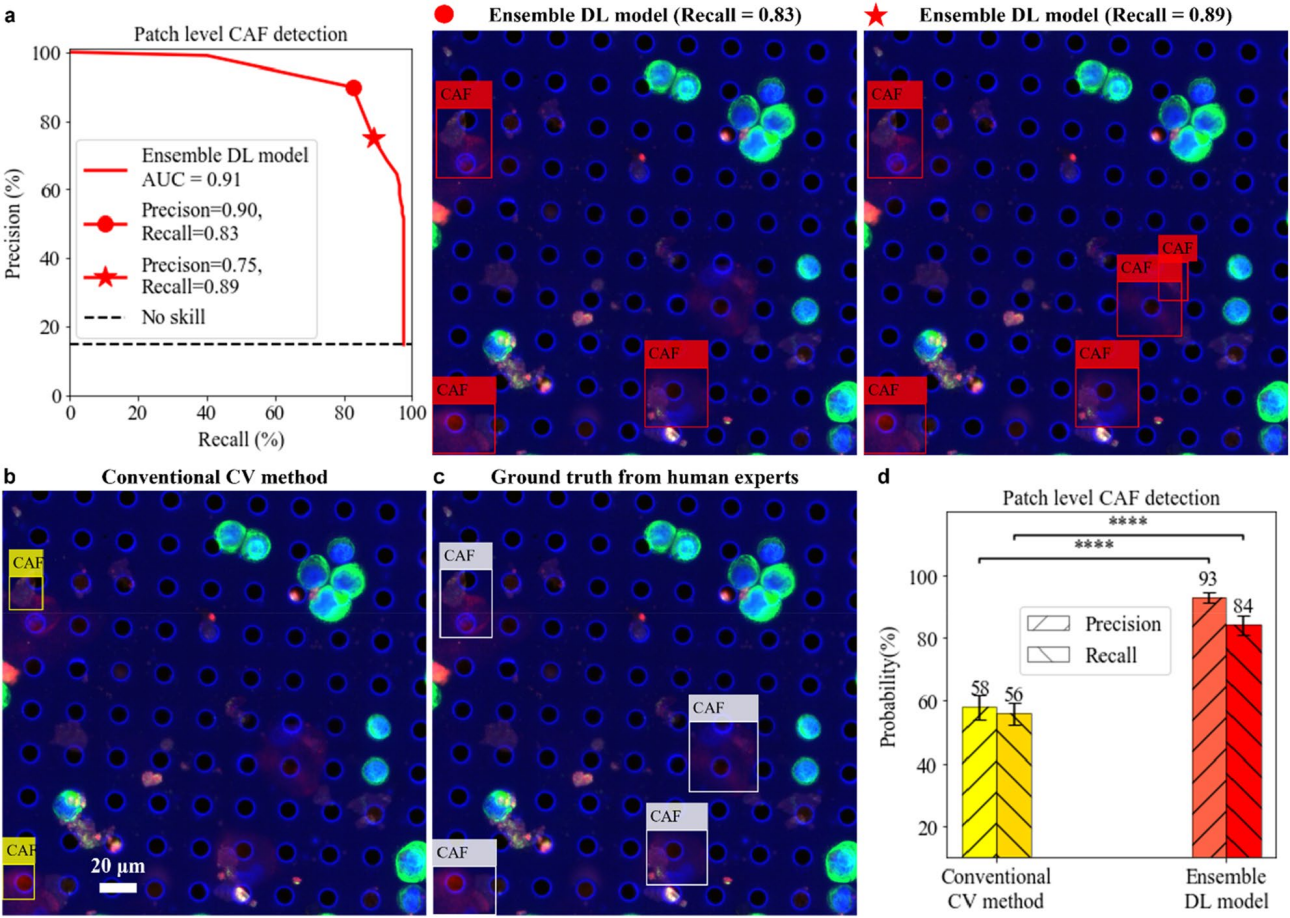
Evaluation of CAF detection. (**a**) Precision-recall curve of the ensemble deep learning (DL) model to detect CAFs in testing patch images. The red dot represents the final chosen operating point. The red star represents another operating point with higher recall but lower precision. Any possible CAF event will be caught but it requires further human analysis to exclude the false alarms. (**b**) CAF detection by conventional computer vision (CV) method. (**c**) Ground truth from human expert annotation. (**d**) Performance comparison between conventional CV method and ensemble DL model to detect CAFs on the patch image level. Both precision and recall of ensemble DL model are significantly higher than the ones of conventional CV method. Statistical analysis uses the ensemble DL model result as the reference to test their difference significance, error bars show standard deviation of precisions and recalls by randomly sampling testing dataset 1000 times and the *p*-values are specified in the figure for *p < 0.05, **p < 0.01, ***p < 0.001, ****p < 0.0001, *NS* not significant, two-sided *z*-test.

We repeated the same training and analysis experiment on CAFs. The results are summarized in Fig. 7. In general, we found CAF detection to be more challenging than mCTC detection for the computational methods as there were more fluorophore precipitates in the specific fluorescence channel for CAF as well as the size and shape of CAFs vary more widely, and as some CAFs were found to be caught within the filter membrane pores and deformed more extensively than the cultured tumor cells. We also note that, within the context of our experiment, the number of CAFs was much lower than that for the mCTCs in our dataset. Under these circumstances, we achieved the final AUC of the precision-recall curve for our ensemble DL CAF detection model of value 0.91. When we seek a balanced precision and recall, this curve yields a precision and recall of 90% and 83%, respectively. Following statistical analysis, as outlined in the "Methods" section, we obtained a final precision and recall of 93% (± 1.7%) and 84% (± 3.1%), respectively, for our DL CAF detection model. In contrast, the conventional CV method gave a balanced precision and recall of 58% (± 3.9%) and 56% (± 3.5%), respectively.

## Discussion

Our microfilter technology provides a robust platform to capture and enrich CTCs and other rare cells of interest from the peripheral blood of cancer patients. Assessments of these events provide opportunities for advanced liquid biopsy diagnostics in a minimally invasive manner. To this end, the combined hardware and deep-learning-based analytical solution that we present here could significantly advance the practice of cell-based, liquid biopsy cancer diagnostics.

We have developed an imaging system that can adequately address the inherent imaging constraints imposed by capturing cells on micropore filters or other surfaces with focus plane variations that are ill-addressed by currently available commercial slide scanners. We showed that our hardware-software hybrid system can locate the correct focal plane within the objective’s FOV at a patch size of 138 μm × 138 μm and it can render AIF whole slide images for our filter with only about 0.7% of the total area missing the target focus. We next showed that this uniform focus quality of the rendering is well suited for DL based object recognition for automated detection of mCTCs and CAFs on the filter. We further demonstrated that the DL approach outperforms conventional CV models in the recognition task. The positive findings form the foundation to begin our next phase of research—collecting clinical CTCs and CAFs samples to train the DL models and apply the trained system for routine automatic identification of CTCs and CAFs in clinical blood samples.

For this study we compared our custom imaging platform with a state-of-the-art scanner, the Olympus VS120. A quantitative analysis on the image focus quality shows that our platform was able to render images with 99.3% of the slide in good-to-moderate focus, while the Olympus VS120 system’s output image resulted in 89.9% of the slide in good-to-moderate focus. Our trained DL model surpasses the conventional computer vision (CV) method in detection accuracy. Our mCTC detection model achieves a precision of 94% (± 0.2%) and a recall of 96% (± 0.2%) and the CAF detection model achieves a precision of 93% (± 1.7%) and a recall of 84% (± 3.1%). For comparison, a conventional CV method based on image processing algorithms following pathologist screening protocol can detect mCTCs with precision of 92% (± 0.2%) and a recall of 78% (± 0.3%) and CAFs with precision of 58% (± 3.9%) and recall of 56% (± 3.5%).

We note the slightly lower AUC value for CAFs detection versus mCTC detection in our experiment. This is likely attributable to the order of magnitude lower CAF instances that we were able to use in this set of experiments. We anticipate that by increasing the number of training instances we will see an improvement in the performance. Empirically, we saw that training instance number on the order of around 5000 resulted in an acceptably high AUC for mCTC analysis. This suggests that we should be able to boost the CAF’s AUC quantity by increasing the training instance to that order of magnitude in our next phase of work. Alternately, we can still employ this existing ensemble model by pivoting its use as a screening tool to aid manual analysis. Specifically, we can choose the operating point to have a high recall (example: recall of 89% and precision of 75% shown in Fig. 7a). This will result in a high identification rate for CAFs with a high incidence of false positives, which can then be subsequently ruled out by the human operator. While a model with slightly lower AUC would still be useful for pre-screening CAFs for skilled observers (i.e., cytopathologists) to make the final assessment, this finding strongly indicates that our clinical research phase should aim to collect more CAFs instances for DL training in order to boost the AUC.

Finally, we conclude by noting that a fully automatic CTC and CAF detection system based on DL techniques is useful for freeing human labor from the tedious cell identification and eliminating human subjectivity from the process. We anticipate that such a system can potentially find applications in clinical research and ultimately in the use of clinical cancer management.

## Methods

### CTC and CAF tissue culture

In this study, we did not use any clinical blood samples from patients. Instead, we used cell lines to mimic the circulating tumor cells and cancer associated fibroblast cells from blood. To create controlled, synthetic cell samples that would roughly mimic the target human cell types expected to be found in clinical blood samples from breast cancer patients, human breast cancer (SKBR3) cells were obtained from the American Type Culture Collection (ATCC) and cultured using phenol red McCoy's 5A medium supplemented with heat-inactivated 10% fetal bovine serum (FBS). The primary CAF23 cell lines were acquired from a collaborator lab (Dr. Dorraya El-Ashry) and have been previously isolated and characterized by her group^54^, which were maintained in phenol red Gibco's Improved Minimum Essential Medium (IMEM) supplemented with heat inactivated 10% FBS. All the cells were grown in Heracell VIOS cell incubators at 37 °C with 5% CO_2_. Cells were passaged continuously by detachment using TrypLE.

### Model system slide preparation

Model systems were created to be utilized for the development of the imaging scanner and cell identification algorithm. SKBR3 and CAF23 cells were cocktailed together and spiked into 7 mL 1X phosphate buffered saline (PBS). The cells were spiked within a range of 500–1000 of each cell type to create a variety of model systems during testing. The spiked cells in 1X PBS were processed using the Circulogix faCTChecker instrument. Post processing the microfilter slides were retrieved from the instrument with the fixed cells captured on the microfilter membrane, ready for downstream immunofluorescence staining and imaging.

### Immunofluorescence staining

SKBR3 and CAF23 cells were detected by double immunostaining using markers that would allow for identification for each cell type, namely pan-cytokeratin (CK) for SKBR3 and fibroblast activation protein (FAP) for CAFs. Samples were blocked for 1 h with blocking buffer consisting of 5% normal goat serum (Life Technologies) and 0.3% Triton X-100 (Sigma Aldrich) in 1X PBS and then incubated overnight at 4ºC with rabbit anti-human FAP (Millipore). Samples were then incubated with a goat anti-rabbit Alexa 594 labeled secondary antibody (Life Technologies) at room temperature for 1 h. Samples were then incubated with Alexa 488 pre-conjugated CK antibody (eBioscience). Samples were counterstained with a combination of DAPI for twelve minutes followed by Hoechst for five minutes, to optimize signal and minimize quenching in the DAPI channel (as it is used as the focus channel), which was experienced during initial testing. Finally, the samples were mounted on coverslips with ProLong Diamond Antifade Mountant (Life Technologies).

### Microscope system and communication software

The whole imaging system is comprised of an inverted epifluorescence microscope (Olympus IX83) with an achromatic objective lens (Olympus PLN 20X, NA 0.4), a motorized lateral scanning stage (Thorlabs MLS203-1 and BBD302) and a high-performance CMOS camera (ISVI IC-M50T-CXP). The control of microscope is through μManager^55^ and a Python wrapper was written on top of it to interface with the microscope. More specifically, it controlled the motorized fluorescent mirror turret, epi-shutter and motorized focus controller of microscope. The lateral scanning stage is controlled by a Python module wrapping Thorlabs APT library^56^ and the camera is communicated with the computer via a Python image acquisition library, Harvester^57^. Finally, all communication functions are assembled in a Python environment.

### Deep learning training and testing

The dataset and code were saved in Google Drive and the training session of all models was run in Google Colab. The assignment of GPU resource was random due to the subscription rules behind Colab. It was either NVIDIA A100 (SXM4, 40 GB) or Tesla V100 (SXM2, 16 GB). The object detection tasks were developed based on an open-source computer vision library, Detectron2^58^, implemented in PyTorch. It provides a large set of baseline results and pre-trained models.

All cell detection models were trained for 1000 epochs and the learning rate was set as 0.00025. The number of output classes was always one and the class label was either mCTC or CAF. Other settings, including optimizer and loss function, were kept as default in the trainer. A training session of 1000 epochs took around 3.5 min to finish on NVIDIA A100 or Tesla V100. We did not observe a significant speed difference probably because our training was not computationally heavy.

### Pathologist screening protocol

The screening criteria to identify mCTCs and CAFs were subjectively based on the fluorescence signal intensity and localization, cell size and shape^28^. A simple decision tree for the process is given in Supplementary Fig. S4. In this project, mCTC clusters were not considered as an independent class but rather as a set of clumped single cells. Once all single mCTCs are detected, we could evaluate their pairwise distance to identify clusters further.

### Statistical analysis

Cell detection results from our ensemble DL method and the conventional CV method were compared using a two-tailed *z*-test of two population proportions with significance classified for *p*-values as: *p < 0.05, **p < 0.01, ***p < 0.001, ****p < 0.0001 and NS, not significant. Statistical analyses were performed using the statistics module in SciPy package with Python (https://docs.scipy.org/doc/scipy/tutorial/stats.html).

To obtain statistically meaningful expectations and standard deviations of precisions and recalls for different detectors, we randomly sampled the testing dataset 1000 times and performed testing on each individual sample. The resulting mean was then reported and used for the two-tailed *z*-test, with the standard deviation being reflected as error bars in Figs. 6 and 7. For patch-level testing, we conducted bootstrapping to sample the original testing patch images. For WSI-level testing, as we only had a single slide available for testing, we randomly cropped 90% of the whole slide area to obtain multiple samples.

## Supplementary Information

Supplementary Information.

## Data Availability

The datasets generated and/or analysed during the current study are available in the Google Drive repository, https://drive.google.com/drive/folders/1hsxoi5tr3_3e-tldonrWFRbi1B7J4Pcz?usp=sharing.

## Code availability

The training and testing code for the ensemble DL mCTC and CAF detection models are available at https://github.com/Scott-Sheen/AI4CTCCAF.

## References

[1] Lambert AW, Pattabiraman DR, Weinberg RA (2017). Emerging biological principles of metastasis. Cell.

[2] Taftaf R, Liu X, Singh S, Jia Y, Dashzeveg NK, Hoffmann AD, El-Shennawy L (2021). ICAM1 initiates CTC cluster formation and trans-endothelial migration in lung metastasis of breast cancer. Nat. Commun..

[3] Plaks V, Koopman CD, Werb Z (2013). Circulating tumor cells. Science.

[4] Williams SCP (2013). Circulating tumor cells. Proc. Natl. Acad. Sci..

[5] Potdar PD, Lotey NK (2015). Role of circulating tumor cells in future diagnosis and therapy of cancer. J. Cancer Metastasis Treatm..

[6] Pimienta M, Edderkaoui M, Wang R, Pandol S (2017). The potential for circulating tumor cells in pancreatic cancer management. Front. Physiol..

[7] Yang C, Chen F, Wang S, Xiong B (2019). Circulating tumor cells in gastrointestinal cancers: Current status and future perspectives. Front. Oncol..

[8] Hofman V, Heeke S, Marquette C-H, Ilié M, Hofman P (2019). Circulating tumor cell detection in lung cancer: But to what end?. Cancers.

[9] Yang Y-P, Giret TM, Cote RJ (2021). Circulating tumor cells from enumeration to analysis: Current challenges and future opportunities. Cancers.

[10] Aceto N, Bardia A, Miyamoto DT, Donaldson MC, Wittner BS, Spencer JA, Yu M (2014). Circulating tumor cell clusters are oligoclonal precursors of breast cancer metastasis. Cell.

[11] Hong Y, Fang F, Zhang Qi (2016). Circulating tumor cell clusters: What we know and what we expect. Int. J. Oncol..

[12] Schuster E, Taftaf R, Reduzzi C, Albert MK, Romero-Calvo I, Liu H (2021). Better together: Circulating tumor cell clustering in metastatic cancer. Trends Cancer.

[13] Ao Z, Shah SH, Machlin LM, Parajuli R, Miller PC, Rawal S, Williams AJ (2015). Identification of cancer-associated fibroblasts in circulating blood from patients with metastatic breast cancer identification of cCAFs from metastatic cancer patients. Cancer Res..

[14] LeBleu VS, Kalluri R (2018). A peek into cancer-associated fibroblasts: origins, functions and translational impact. Dis. Models Mech..

[15] Sahai E, Astsaturov I, Cukierman E, DeNardo DG, Egeblad M, Evans RM, Fearon D (2020). A framework for advancing our understanding of cancer-associated fibroblasts. Nat. Rev. Cancer.

[16] Ping Q, Yan R, Cheng X, Wang W, Zhong Y, Hou Z, Shi Y, Wang C, Li R (2021). Cancer-associated fibroblasts: Overview, progress, challenges, and directions. Cancer Gene Ther..

[17] Boya M, Ozkaya-Ahmadov T, Swain BE, Chu C-H, Asmare N, Civelekoglu O (2022). Ruxiu Liu et al. High throughput, label-free isolation of circulating tumor cell clusters in meshed microwells. Nat. Commun..

[18] Patil P, Kumeria T, Losic D, Kurkuri M (2015). Isolation of circulating tumour cells by physical means in a microfluidic device: A review. RSC Adv..

[19] Chen L, Bode AM, Dong Z (2017). Circulating tumor cells: Moving biological insights into detection. Theranostics.

[20] Maertens Y, Humberg V, Erlmeier F, Steffens S, Steinestel J, Bögemann M, Schrader AJ, Bernemann C (2017). Comparison of isolation platforms for detection of circulating renal cell carcinoma cells. Oncotarget.

[21] Vona G, Sabile A, Louha M, Sitruk V, Romana S, Schütze K, Capron F (2000). Isolation by size of epithelial tumor cells: A new method for the immunomorphological and molecular characterization of circulating tumor cells. Am. J. Pathol..

[22] Zheng S, Lin H, Liu J-Q, Balic M, Datar R, Cote RJ, Tai Y-C (2007). Membrane microfilter device for selective capture, electrolysis and genomic analysis of human circulating tumor cells. J. Chromatogr. A.

[23] Desitter I, Guerrouahen BS, Benali-Furet N, Wechsler J, Jänne PA, Kuang Y, Yanagita M (2011). A new device for rapid isolation by size and characterization of rare circulating tumor cells. Anticancer Res..

[24] Seal SH (1959). Silicone flotation: A simple quantitative method for the isolation of free-floating cancer cells from the blood. Cancer.

[25] Gertler, R., Rosenberg, R., Fuehrer, K., Dahm, M., Nekarda, H., & Siewert, J.R. Detection of circulating tumor cells in blood using an optimized density gradient centrifugation. In* Molecular Staging of Cancer*, pp. 149–155. Springer, Berlin, Heidelberg (2003). 10.1007/978-3-642-59349-9_13 12790329

[26] Shahneh FZ (2013). Sensitive antibody-based CTCs detection from peripheral blood. Hum. Antibodies.

[27] Yang C, Zou K, Yuan Z, Guo T, Xiong B (2018). Prognostic value of circulating tumor cells detected with the Cell Search System in patients with gastric cancer: Evidence from a meta-analysis. Onco. Targets. Ther..

[28] Mansilla C, Soria E, Ramírez N (2018). The identification and isolation of CTCs: A biological Rubik’s cube. Crit. Rev. Oncol. Hematol..

[29] Xu Yu, Qin T, Li J, Wang X, Gao C, Chao Xu, Hao J, Liu J, Gao S, Ren He (2017). Detection of circulating tumor cells using negative enrichment immunofluorescence and an in situ hybridization system in pancreatic cancer. Int. J. Mol. Sci..

[30] Guo W, Yang XR, Sun YF, Shen MN, Ma XL, Wu J, Zhang CY (2014). Clinical significance of EpCAM mRNA-positive circulating tumor cells in hepatocellular carcinoma by an optimized negative enrichment and qRT-PCR–based platform. Clin. Cancer Res..

[31] Heitzer E, Auer M, Gasch C, Pichler M, Ulz P, Hoffmann EM, Lax S (2013). Complex tumor genomes inferred from single circulating tumor cells by array-CGH and next-generation sequencing CTC analysis by array-CGH and next-generation sequencing. Cancer Res..

[32] Sha M, Jeong S, Qiu B-J, Tong Y, Xia L, Ning Xu, Zhang J-J, Xia Q (2018). Isolation of cancer-associated fibroblasts and its promotion to the progression of intrahepatic cholangiocarcinoma. Cancer Med..

[33] Jiang R, Agrawal S, Aghaamoo M, Parajuli R, Agrawal A, Lee AP (2021). Rapid isolation of circulating cancer associated fibroblasts by acoustic microstreaming for assessing metastatic propensity of breast cancer patients. Lab. Chip.

[34] Williams AJ, Chung J, Xiaoze Ou, Zheng G, Rawal S, Ao Z, Datar R, Yang C, Cote RJ (2014). Fourier ptychographic microscopy for filtration-based circulating tumor cell enumeration and analysis. J. Biomed. Opt..

[35] Kohlberger T (2019). Whole-slide image focus quality: Automatic assessment and impact on AI cancer detection. J. Pathol. Inf..

[36] Svensson CM, Krusekopf S, Lücke J, Figge MT (2014). Automated detection of circulating tumor cells with naive Bayesian classifiers. Cytometry A.

[37] Svensson C-M, Hübler R, Figge MT (2015). Automated classification of circulating tumor cells and the impact of interobsever variability on classifier training and performance. J. Immunol. Res..

[38] Lannin TB, Thege FI, Kirby BJ (2016). Comparison and optimization of machine learning methods for automated classification of circulating tumor cells. Cytometry A.

[39] Stevens M, Nanou A, Terstappen LWMM, Driemel C, Stoecklein NH, Coumans FAW (2022). StarDist image segmentation improves circulating tumor cell detection. Cancers.

[40] Mao, Y., Yin, Z., & Schober, J. A deep convolutional neural network trained on representative samples for circulating tumor cell detection. In *2016 IEEE Winter Conference on Applications of Computer Vision (WACV)*, pp. 1–6. IEEE (2016).

[41] Zeune LL, Boink YE, Dalum G, Nanou A, de Wit S, Andree KC, Swennenhuis JF, van Gils SA, Terstappen LWMM, Brune C (2020). Deep learning of circulating tumour cells. Nat. Mach. Intell..

[42] Boecker, W., Rolf, W., Muller, W.-U., & Streffer, C. Autofocus algorithms for fluorescence microscopy. In *Applications of Digital Image Processing XIX*, **2847**, pp. 445–456. SPIE, (1996).

[43] Peng T, Thorn K, Schroeder T, Wang L, Theis FJ, Marr C, Navab N (2017). A BaSiC tool for background and shading correction of optical microscopy images. Nat. Commun..

[44] Yang SJ, Berndl M, Ando DM, Barch M, Narayanaswamy A, Christiansen E, Hoyer S (2018). Assessing microscope image focus quality with deep learning. BMC Bioinf..

[45] Bankhead P, Loughrey MB, Fernández JA, Dombrowski Y, McArt DG, Dunne PD, McQuaid S (2017). QuPath: Open source software for digital pathology image analysis. Sci. Rep..

[46] Lin, T.-Y., Maire, M., Belongie, S., Hays, J., Perona, P., Ramanan, D., Dollár, P., & Zitnick, C. L. Microsoft coco: Common objects in context. In *European conference on computer vision*, pp. 740–755. Springer, Cham (2014).

[47] Liu Li, Ouyang W, Wang X, Fieguth P, Chen J, Liu X, Pietikäinen M (2020). Deep learning for generic object detection: A survey. Int. J. Comput. Vision.

[48] Zhao Z-Q, Zheng P, Shou-tao Xu, Xindong Wu (2019). Object detection with deep learning: A review. IEEE Trans. Neural Netw. Learn. Syst..

[49] Lin, T.-Y., Goyal, P., Girshick, R., He, K., & Dollár, P. Focal loss for dense object detection. In *Proceedings of the IEEE international conference on computer vision*, pp. 2980–2988 (2017).

[50] Ren S, He K, Girshick R, Sun J (2015). Faster r-cnn: Towards real-time object detection with region proposal networks. Adv. Neural Inf. Process. Syst..

[51] He, K., Zhang, X., Ren, S., & Sun, J. Deep residual learning for image recognition. In *Proceedings of the IEEE conference on computer vision and pattern recognition*, pp. 770–778. (2016).

[52] Xie, S., Girshick, R., Dollár, P., Tu, Z., & He, K. Aggregated residual transformations for deep neural networks. In *Proceedings of the IEEE conference on computer vision and pattern recognition*, pp.1492–1500. (2017).

[53] Everingham M, Gool LV, Williams CKI, Winn J, Zisserman A (2010). The pascal visual object classes (VOC) challenge. Int. J. Comput. Vis..

[54] Drews-Elger K, Brinkman JA, Miller P, Shah SH, Harrell JC, Da Silva TG, Ao Z (2014). Primary breast tumor-derived cellular models: characterization of tumorigenic, metastatic, and cancer-associated fibroblasts in dissociated tumor (DT) cultures. Breast Cancer Res. Treatm..

[55] Edelstein AD, Tsuchida MA, Amodaj N, Pinkard H, Vale RD, Stuurman N (2014). Advanced methods of microscope control using μManager software. J. Biol. Methods.

[56] https://github.com/qpit/thorlabs_apt.

[57] https://github.com/genicam/harvesters.

[58] https://github.com/facebookresearch/detectron2.

