# Supplementary material for: Automatic detection of circulating tumor cells and cancer associated fibroblasts using deep learning
Source: Sci Rep. 2023 Apr 7;13:5708. doi: 10.1038/s41598-023-32955-0 (PMC10082202; doi:10.1038/s41598-023-32955-0)
Supplement: Supplementary file 1 — Supplementary Information. [file 41598_2023_32955_MOESM1_ESM.docx]

**Supplementary Information**

**Automatic Detection of Circulating Tumor Cells and**

**Cancer Associated Fibroblasts using Deep Learning**

Cheng Shen^1^, Siddarth Rawal^2^, Rebecca Brown^2^, Haowen Zhou^1^, Ashutosh Agarwal^3^, Mark A. Watson^2^, Richard J. Cote^2*^, Changhuei Yang^1*^

^1^ Department of Electrical Engineering, California Institute of Technology, Pasadena, CA 91125, USA

^2^ Department of Pathology and Immunology, Washington University School of Medicine, St. Louis, MO 63110, USA

^3^ Department of Biomedical Engineering, DJTMF Biomedical Nanotechnology Institute, University of Miami, Coral Gables, FL 33146, USA

^*^Co-corresponding authors. Email: chyang@caltech.edu; rcote@wustl.edu

**Including:**

Supplementary figures 1 to 8


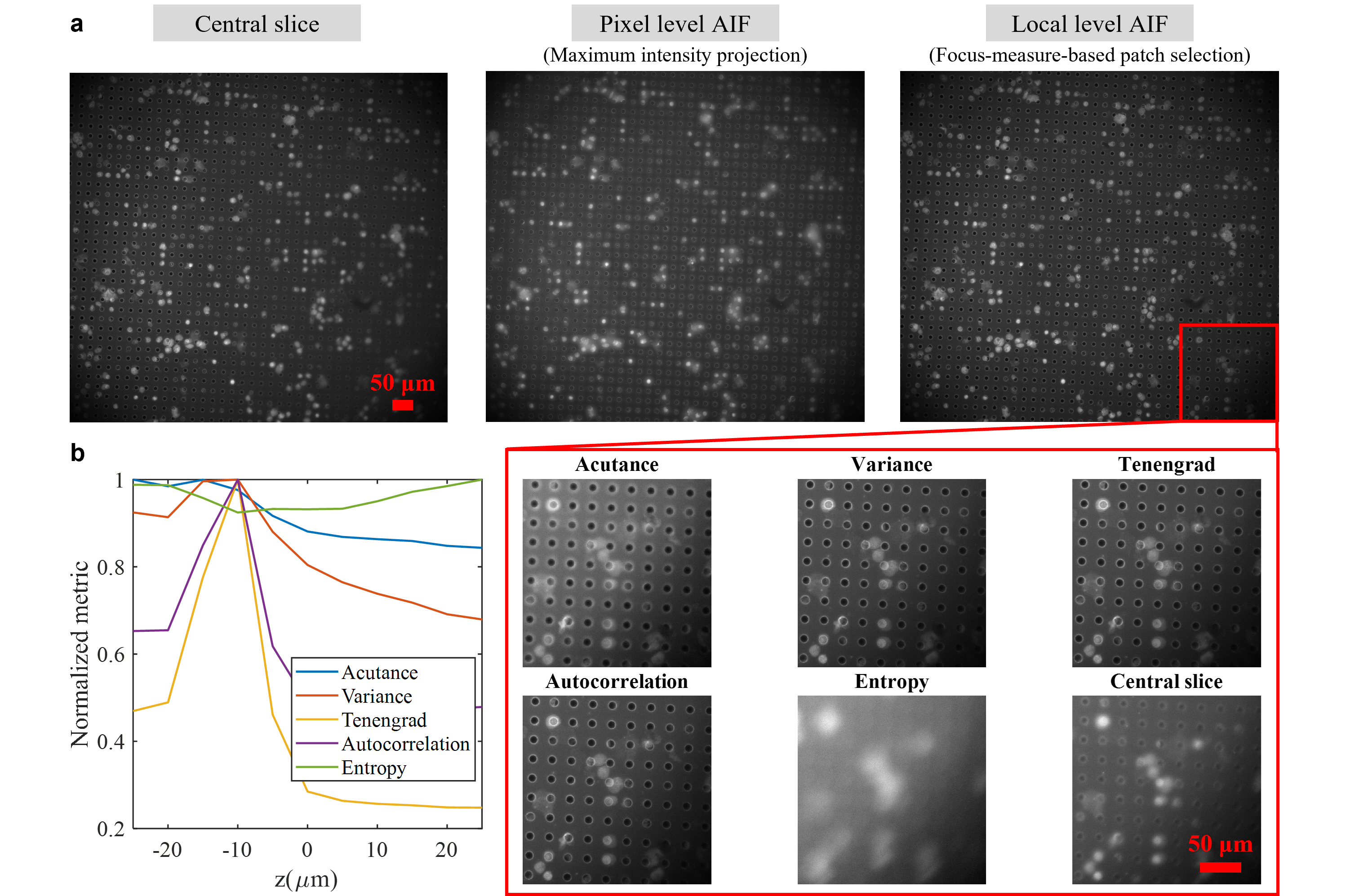


**Supplementary Figure 1. Comparison of different all-in-focus (AIF) compression methods.**

**a.** Central slice of a *z*-stack from DAPI channel, compared with pixel level AIF compression result based on maximum intensity projection and local patch level AIF compression results based on different *F*-metrics. **b.** Normalized curve of different focus measures versus *z* positions.

We can see from the central slice that a single z-slice is not capable of presenting all cells in good focus. Maximum intensity projection, where we project the maximum pixel value along the z-axis, is one possible way to resolve this. However, as can see from the image, this simply creates an image with significant haze artifacts. Local-level patch selection AIF methods can avoid this problem as long as the focus measure is correctly chosen. Here, Tenegrad function is chosen as our metric because it can find the correct focus *z* position with excellent sensitivity.


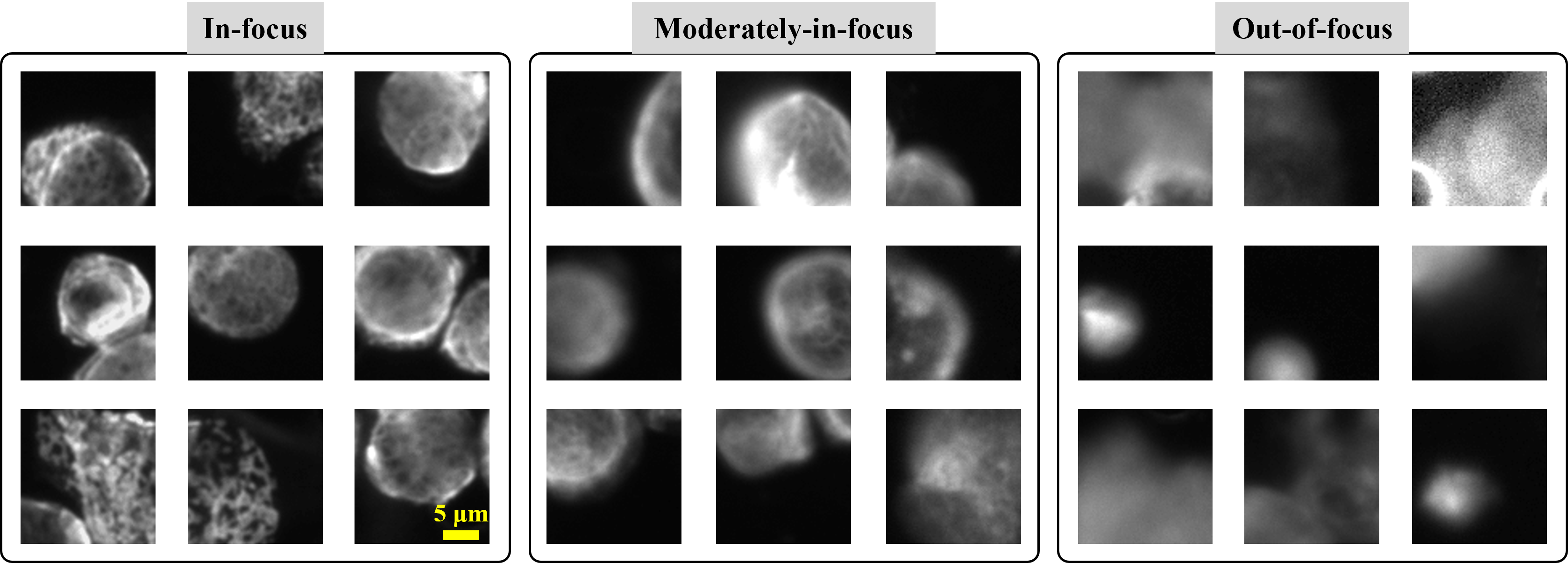


**Supplementary Figure 2. Three focus quality classes derived from a reported absolute measure method.**

To compare the focus quality of our custom slide scanner and the commercial Olympus slide scanner quantitatively, we used a reported absolute focus quality measure method based on deep learning [Ref. 44]. This method was trained on wide field fluorescence images of U2OS cells with nuclei stained by Hoechst and has been proven to generalize well to other stains, such as Tubulin and Phalloidin, and the unseen MCF-7 cancer cell type. As our images are also wide field fluorescence images of cancer cells, we expected this method to generalize well to our data. To ensure the predicted focus quality metrics aligned with human visual evaluation, we verified their classification results and found them to be in good agreement. Patch images classified as "in-focus" displayed clear cell cytokeratin details, while "moderately-in-focus" images still appeared highly likely to be cell cytokeratin. However, "out-of-focus" images barely resembled cells and might appear as precipitates, shown in some examples here.


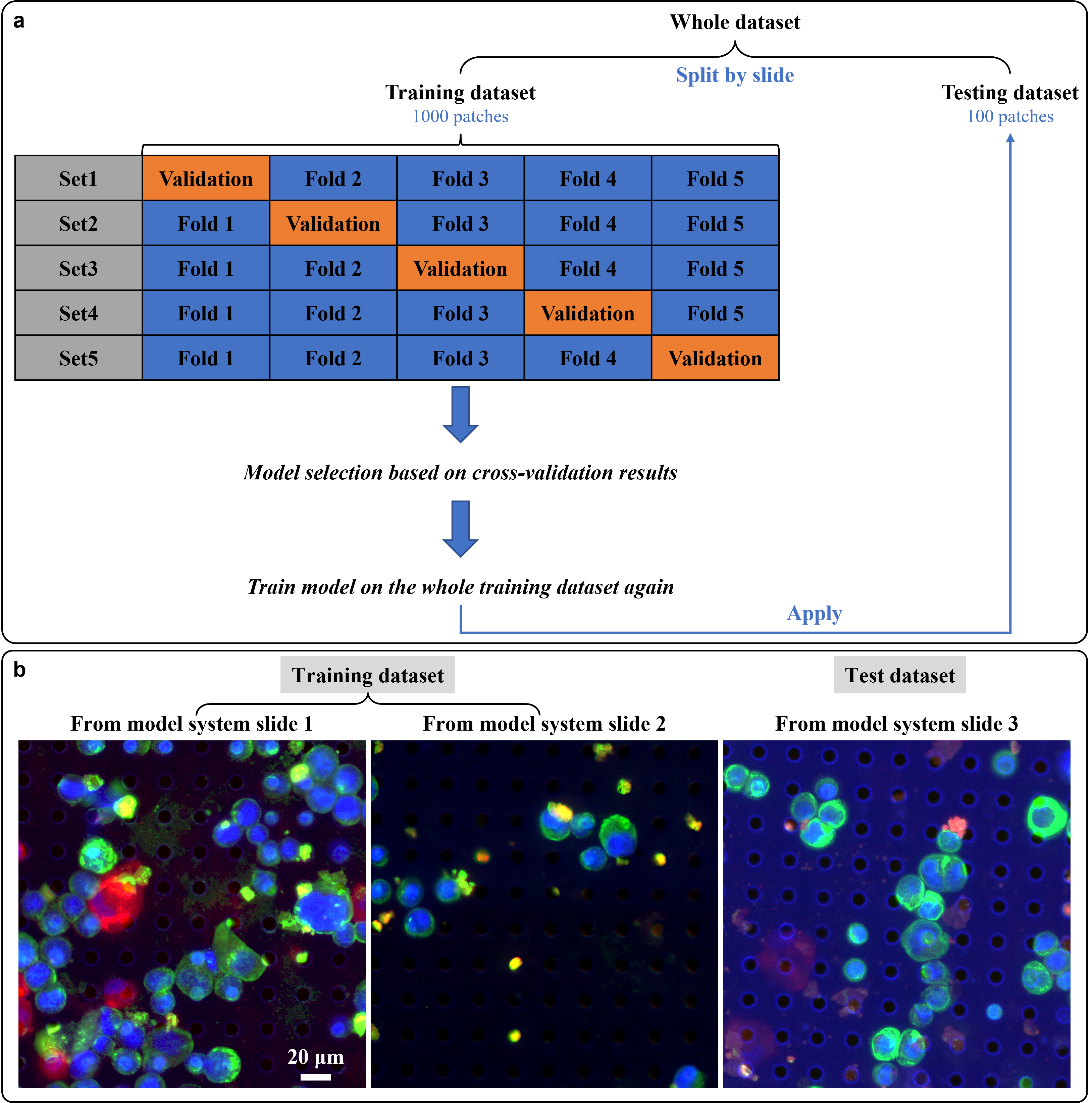


**Supplementary Figure 3. Overall plan of data splitting for deep learning.**

**a.** The dataset was initially split into a training dataset and a testing dataset by slide. Two out of the three slides from different batches were used for training, while the remaining slide was used for testing. During the deep learning model training, we conducted 5-fold cross-validation to select the best model architecture. Subsequently, the model was retrained using the entire training dataset for final testing. **b.** To reduce color variation, we manually adjusted the histogram of each color channel in every whole slide image so that cells of the same type displayed a consistent color pattern before inputting them into the deep learning model. Despite this, the background varied across different slides, with microfilter holes producing autofluorescence in the DAPI channel being particularly pronounced in slide 3. However, the high precision values obtained from our trained deep learning model during testing indicate that it was not affected by this background 'noise'.


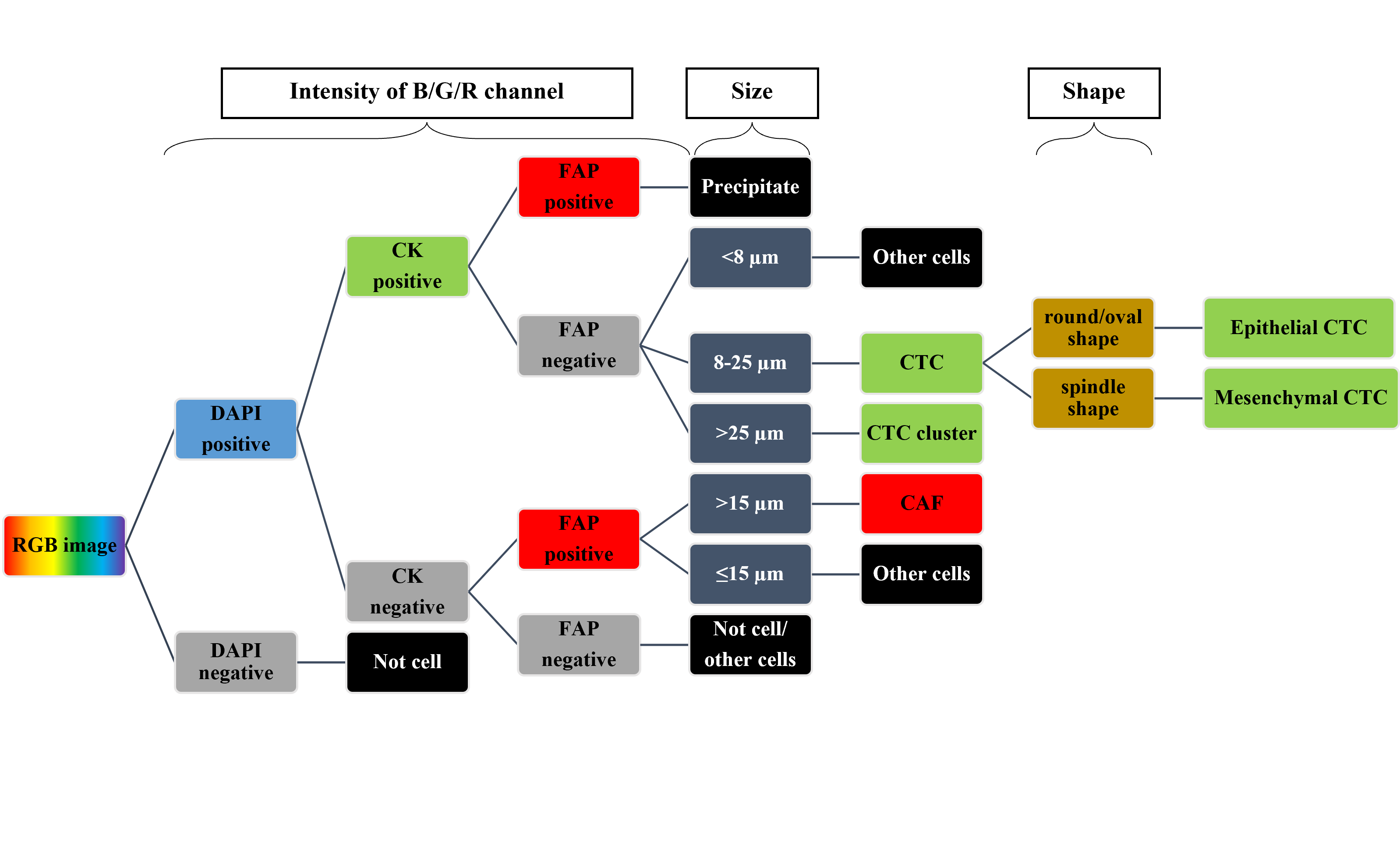


**Supplementary Figure 4. Pathology screening decision tree.**

The screening of CTCs and CAFs from RGB fluorescence images relies on the fluorescence signal intensity and location as well as cell size and shape. The main goal is to distinguish them from other cells, precipitate, and junk. CTC cluster can be seen as a collection of single CTCs from the perspective of computer vision. Also, single CTCs could be further classified into two subtypes, which could be a future work, based on our current results with excellent mCTC detection accuracy. CK, cytokeratin; FAP, fibroblast activation protein.


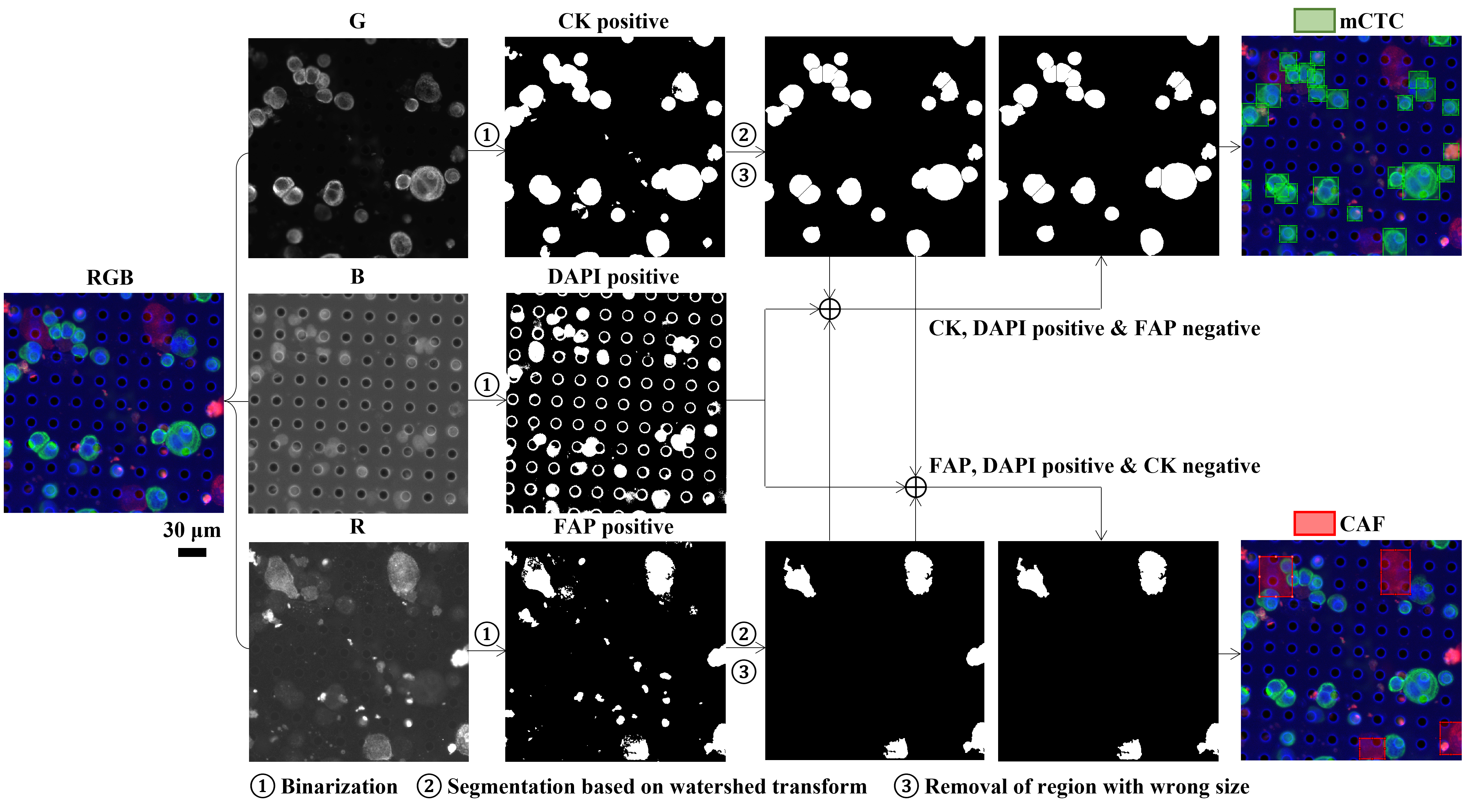


**Supplementary Figure 5. Conventional computer vision method for mCTC and CAF detection using image processing algorithms.**

The conventional computer vision method to detect mCTCs and CAFs from RGB fluorescence images was designed based on the pathology screening protocol shown in Supplementary Fig. 4. First, three channels were separately binarized to get a bunch of positive events, DAPI positive (blue channel), CK positive (green channel) and FAP positive (red channel). Then, CK and FAP positive events were segmented using watershed transform and cleaned by removing event regions with size below or beyond expectations. Finally, binary images from three channels were cross-checked. The event regions which were DAPI and CK positive but FAP negative were assumed to be mCTCs and those which were DAPI and FAP positive but CK negative were taken as CAFs. Here, DAPI positive events got confirmed by calculating mean intensity value within the area of CK/FAP positive events. This approach helped reject most of the microfilter holes due to their hollow structure. During this image processing pipeline, the binarization threshold, segmentation parameters and size threshold were all chosen and optimized by human.


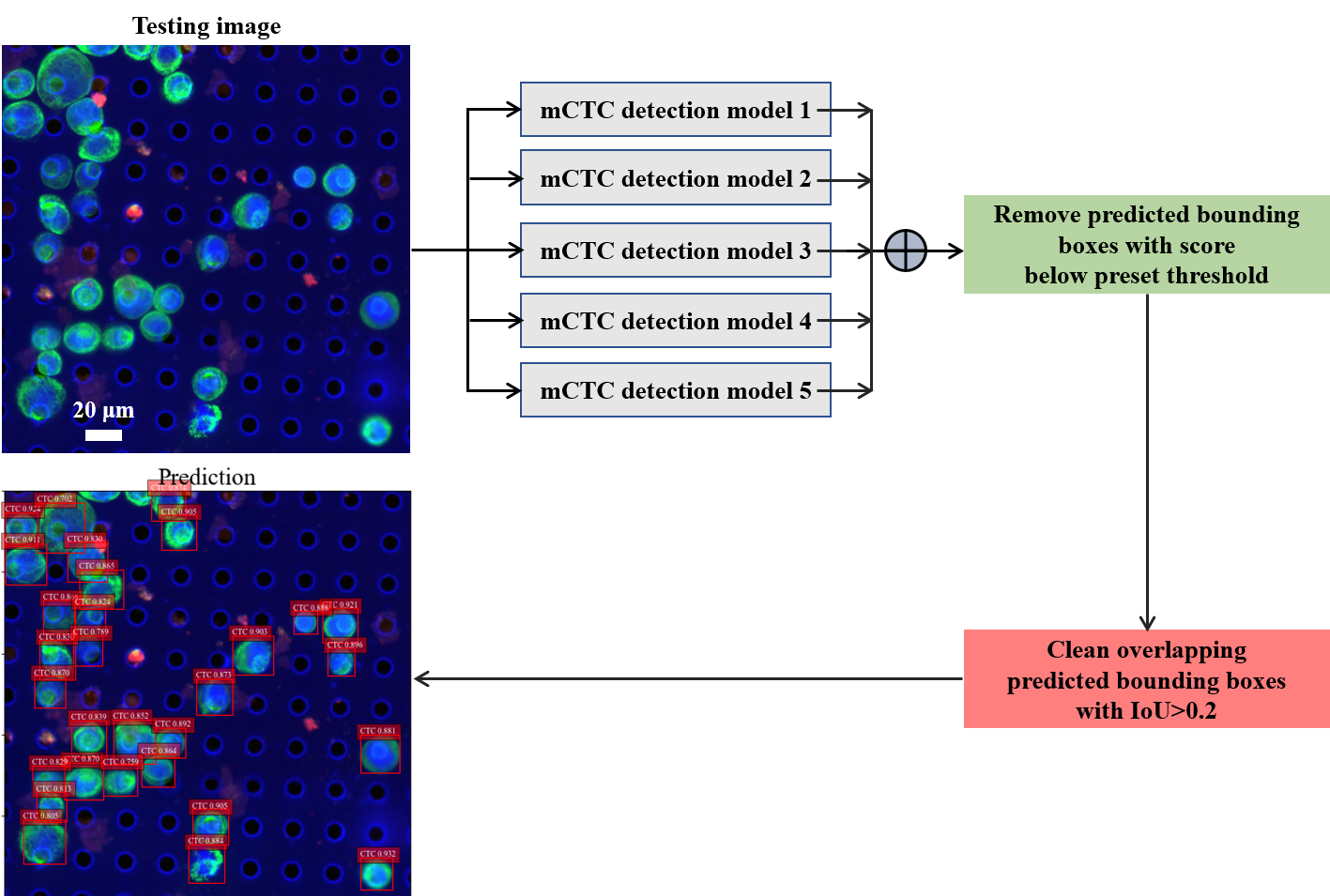


**Supplementary Figure 6. Ensemble five object detection models and cleaning of prediction result.**

Five cell detection models have the same neural network architecture but are trained on randomized image batches. The ensembled prediction is a set of bounding boxes with confidence score. We first remove the predicted boxes with score lower than a chosen threshold. Then, the repeated boxes predicted for the same cell can be reduced to a single box by quantifying the pairwise overlapping ratio between boxes with intersection-over-union (IoU) metric, finding the clustered boxes and only keeping the one with the highest score.


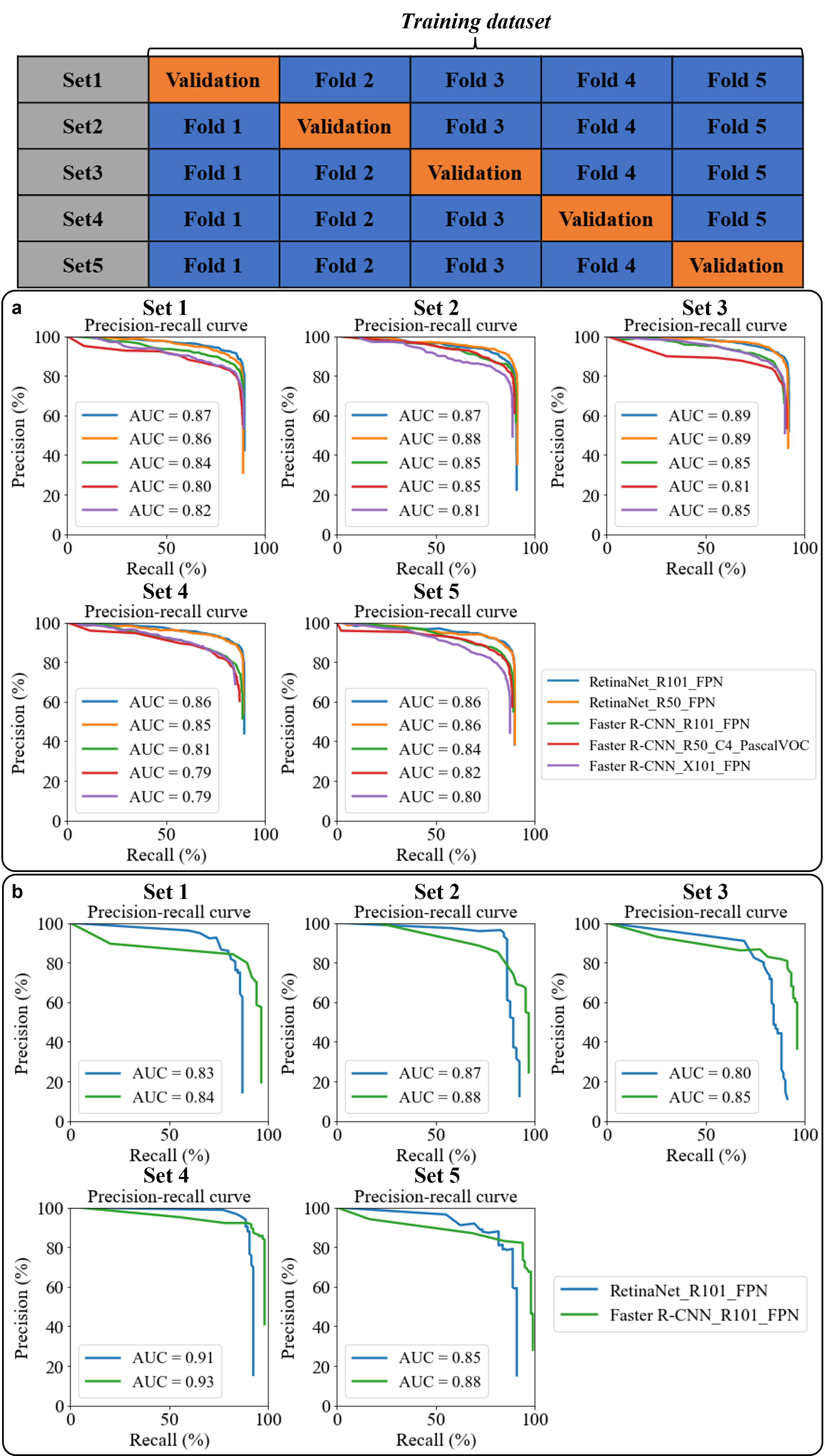


**Supplementary Figure 7. Comparison of different object detection baseline models by 5-fold cross validation.**

**a.** mCTC detection by five selected detectors differing in either network architecture, backbone or pretraining benchmark dataset. From the precision-recall curves, RetinaNet and Faster R-CNN with ResNet-101 backbone pretrained on COCO dataset achieved the best area under curve (AUC) in one-stage and two-stage detectors, respectively. **b.** Only the best one-stage and two-stage models were tested on the CAF detection task. The two-stage detector, Faster R-CNN, had better localization and recognition accuracy as indicated by higher AUC.


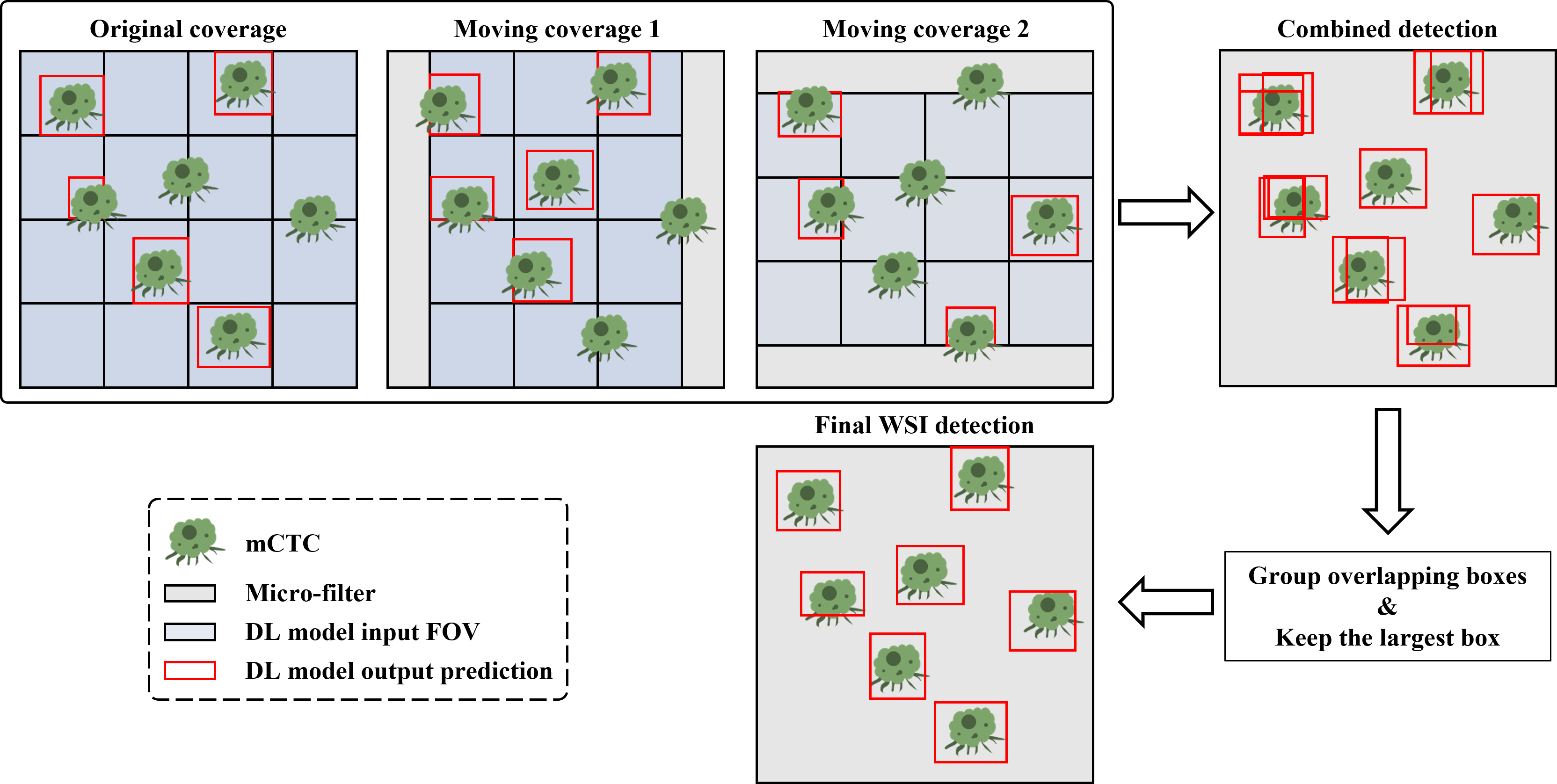


**Supplementary Figure 8. Cell detection at the whole slide image level.**

Any detector has a limited input field of view (FOV). When cells are located across the boundary between two FOVs, only partial cells are visible, therefore they would likely be ignored by the detectors. To solve this issue, we can move the input FOVs covering the whole slide by half size of FOV side length horizontally and vertically, as shown above. In this way, the cells across the boundary could be fully covered and detected. We then use the same strategy shown in Supplementary Fig. 6 to eliminate overlapping boxes by calculating pairwise IoU metrics, grouping them and keeping the largest one. Final WSI detection results show good match with the ground truth.
